# Intelligent scheduling and resource allocation for urban air mobility networks based on graph neural networks

**DOI:** 10.1038/s41598-026-48143-9

**Published:** 2026-04-17

**Authors:** Xiaoqi Xu, Yiyang Zhao, Peilong Zhang

**Affiliations:** 1 School of Law and Business, Shaoyang University, Shaoyang, 422000 Hunan China; 2 Institute of Internet Industry, Tsinghua University, Beijing, 100000 China

**Keywords:** Urban air mobility, Graph neural network, Intelligent scheduling, Resource allocation, Spatiotemporal attention, Low-altitude economy, Engineering, Mathematics and computing

## Abstract

Urban Air Mobility (UAM) represents a promising solution to metropolitan transportation challenges, yet efficient scheduling and resource allocation in large-scale UAM networks remain computationally intractable for traditional optimization methods. This paper proposes a hybrid scheduling framework that couples graph neural network prediction with deterministic constraint repair for UAM scheduling and resource allocation. We first develop a heterogeneous graph representation scheme that captures diverse network elements including vertiports, waypoints, and aircraft with their distinct attributes. A spatiotemporal graph attention network (ST-GAT) architecture is then constructed, integrating spatial topology awareness with temporal dynamics modeling through dual attention channels. Furthermore, an adaptive multi-objective optimization strategy is designed to balance throughput maximization, delay minimization, and energy efficiency while satisfying operational constraints. Experimental results on a simulated metropolitan UAM network demonstrate that the proposed method achieves 94.2% throughput under peak demand conditions with average delay of 4.1 min, outperforming genetic algorithm and simulated annealing baselines by 15.9% and 19.9% respectively. The integrated pipeline—comprising neural scheduling prediction and post-processing feasibility repair—maintains sub-second inference latency up to 250 concurrent aircraft and 60 vertiports, confirming its suitability for real-time operational deployment within the tested scale.

## Introduction

 Urban Air Mobility (UAM) has emerged as a transformative paradigm in metropolitan transportation systems, fundamentally reshaping how passengers and cargo move through congested urban environments. The global aviation community has witnessed unprecedented interest in this domain, driven primarily by mounting traffic congestion in megacities and the pressing demand for sustainable transportation alternatives^1^. Unlike conventional ground-based transit solutions, UAM operates within low-altitude airspace, offering a three-dimensional mobility framework that bypasses terrestrial infrastructure constraints. This nascent industry has attracted substantial investment from aerospace manufacturers, technology firms, and governmental agencies worldwide, reflecting widespread confidence in its commercial viability and societal impact^2^.

The low-altitude economy represents a burgeoning sector that encompasses diverse applications ranging from passenger transport to logistics delivery and emergency medical services. Electric Vertical Take-Off and Landing (eVTOL) aircraft stand at the core of this economic transformation, offering quieter operations, reduced emissions, and enhanced maneuverability compared to traditional rotorcraft^3^. Major aerospace corporations and innovative startups have accelerated their eVTOL development programs, with several prototypes already completing certification test flights. Projections from industry analysts suggest that the global UAM market could exceed hundreds of billions of dollars within the next decade, contingent upon successful regulatory approval and public acceptance^4^. China has demonstrated particular enthusiasm for low-altitude economy development, establishing dedicated policy frameworks and designating pilot cities for UAM implementation^5^.

Research efforts addressing UAM scheduling and resource allocation have proliferated across academic institutions in recent years. International scholars have explored various optimization approaches, including mixed-integer programming formulations for vertiport capacity management and dynamic routing algorithms for on-demand air taxi services^6^. European research consortia have investigated airspace integration challenges, proposing segregated corridors and geofencing mechanisms to ensure safe coexistence with manned aviation^7^. Domestic researchers have contributed meaningfully to this field as well, developing demand prediction models and examining infrastructure deployment strategies tailored to Chinese urban contexts^8^.

Recent advances in resource optimization for aerial networks have introduced novel paradigms worth examining. Chen et al. proposed a multi-objective resource optimization framework for UAV-enabled heterogeneous cellular networks, combining serverless federated learning with power-domain NOMA to achieve efficient spectrum and energy allocation^9^. Their work demonstrates how distributed learning architectures can handle complex multi-objective trade-offs in dynamic aerial environments. Similarly, Zhang et al. developed a dynamic offloading strategy in mobile edge computing environments, incorporating traffic-aware network slicing with adaptive TD3 reinforcement learning to optimize computational resource distribution^10^. This approach reveals the potential of combining network slicing techniques with deep reinforcement learning for real-time resource management. Wang et al. further extended these concepts through hierarchical federated learning for adaptive offloading in multi-access edge networks, enabling real-time system adaptation without centralized coordination^11^. These studies collectively highlight emerging trends toward distributed intelligence and adaptive optimization in aerial and edge computing systems.

Nevertheless, existing methodologies predominantly treat UAM networks as conventional optimization problems, neglecting the inherent topological characteristics that distinguish airspace networks from surface transportation systems. Current approaches fail to capture the complex interdependencies among airspace nodes, and few studies have attempted to integrate graph-based representations with spatiotemporal attention mechanisms for joint scheduling and resource allocation.

Traditional scheduling approaches encounter significant limitations when confronted with large-scale dynamic UAM networks. Centralized optimization methods suffer from combinatorial explosion as fleet sizes and vertiport numbers increase, rendering real-time decision-making computationally intractable^12^. Heuristic algorithms, while faster, frequently converge to suboptimal solutions and struggle to adapt when traffic patterns shift unexpectedly. Perhaps more critically, conventional techniques fail to capture the complex interdependencies among airspace nodes, treating each vertiport or waypoint as an isolated entity rather than an interconnected element within a broader network topology. Such reductionist perspectives overlook crucial relational information that could inform more effective scheduling decisions^13^. The inability to model spatial correlations and propagate contextual information across network components represents a fundamental methodological gap.

Graph Neural Networks (GNNs) present a compelling solution to these shortcomings, offering architectural advantages uniquely suited to networked transportation systems. By representing UAM infrastructure as graph structures where vertiports constitute nodes and flight corridors form edges, GNNs can naturally encode spatial relationships and enable information propagation across topologically connected elements^14^. The message-passing mechanism inherent to GNN architectures allows each node to aggregate features from neighboring nodes, thereby learning representations that capture both local characteristics and global network context. This capability proves particularly valuable for UAM scheduling, where decisions at one vertiport inevitably influence operations at connected facilities. Furthermore, trained GNN models can generate scheduling decisions with minimal inference latency, addressing the real-time responsiveness requirements that deterministic optimization methods cannot satisfy^15^.

This paper presents a comprehensive investigation into GNN-enabled intelligent scheduling and resource allocation for UAM networks. The research encompasses three interrelated components: first, establishing a graph-based modeling framework that represents UAM operational networks with appropriate node and edge feature engineering; second, constructing GNN architectures specifically designed for multi-objective scheduling tasks including departure sequencing, route assignment, and conflict resolution; third, developing resource allocation optimization strategies that balance vertiport capacity utilization, fleet distribution, and energy consumption. The principal contributions of this work include a novel heterogeneous graph representation scheme capturing diverse UAM network elements, an attention-enhanced GNN model achieving superior scheduling performance under varying demand scenarios, and an integrated optimization framework enabling joint scheduling and resource allocation decisions. These innovations collectively advance the methodological foundation for intelligent UAM network management.

## Theoretical foundation and technical framework

### Urban air mobility network modeling theory

A UAM system comprises three fundamental infrastructure elements that collectively enable aerial transportation services within metropolitan regions. Vertiports serve as ground-based facilities where eVTOL aircraft conduct takeoff and landing operations, ranging from small rooftop pads accommodating single vehicles to large multi-gate terminals processing dozens of simultaneous movements^16^. Air corridors constitute predefined flight pathways connecting vertiports, designed to maintain safe separation from obstacles and other airspace users while minimizing noise exposure to communities below. The eVTOL fleet itself represents the mobile component, with individual aircraft characterized by passenger capacity, cruise speed, energy consumption rates, and operational range constraints^17^.

Graph theory provides an intuitive yet mathematically rigorous framework for representing UAM network topology. We define the UAM network as a directed graph $\:G=(V,E)$, where the vertex set $\:V$ encompasses all vertiports and the edge set $\:E$ contains permissible flight corridors connecting vertex pairs. Each node $\:{v}_{i}\in\:V$ carries an attribute vector encoding facility-specific properties, formally expressed as:

1 $$\:{x}_{i}=[{c}_{i},{\lambda\:}_{i},{\mu\:}_{i},{e}_{i},{p}_{i}{]}^{T}$$

where $\:{c}_{i}$ denotes pad capacity, $\:{\lambda\:}_{i}$ represents arrival demand intensity, $\:{\mu\:}_{i}$ indicates service rate, $\:{e}_{i}$ captures available charging infrastructure, and $\:{p}_{i}$ specifies geographic coordinates^18^. Edge attributes similarly encode corridor characteristics through the formulation:

2 $$\:{e}_{ij}=[{d}_{ij},{t}_{ij},{\omega\:}_{ij},{\kappa\:}_{ij}{]}^{T}$$

encompassing distance $\:{d}_{ij}$, nominal travel time $\:{t}_{ij}$, capacity $\:{\omega\:}_{ij}$, and weather sensitivity coefficient $\:{\kappa\:}_{ij}$^19^.

UAM networks exhibit pronounced spatiotemporal dynamics that distinguish them from static graph structures. Demand patterns fluctuate considerably across daily periods, with morning and evening peaks mirroring ground commuter behavior^20^. The network state evolves continuously as aircraft traverse corridors, vertiport queues build and dissipate, and weather conditions alter corridor availability. We capture this temporal evolution through a time-indexed graph representation:

3 $$\:G\left(t\right)=(V,E,X(t),A(t\left)\right)$$

where $\:X\left(t\right)$ aggregates all node feature vectors and $\:A\left(t\right)$ represents the weighted adjacency matrix at timestamp $\:t$^21^. This formulation establishes the mathematical foundation upon which subsequent GNN architectures operate.

To clarify the information architecture underlying our system model, we specify the data sources, ownership, and update protocols for each information type. Table 1 summarizes these specifications. Vertiport state information, including queue lengths and pad availability, originates from ground-based facility management systems with update intervals of 10 s. Aircraft position and velocity data derive from ADS-B transponders broadcasting at 1-second intervals, while battery state-of-charge readings transmit through dedicated telemetry links every 5 s. Corridor availability status comes from the airspace management authority and updates upon weather changes or regulatory modifications, typically at 5-minute intervals during normal operations. Demand forecasts aggregate from booking platforms and historical patterns, refreshing every 5 min to capture emerging travel requests.

**Table 1:** Information architecture and update specifications.

Information type	Data source	Update frequency	Ownership
Vertiport queue length	Ground facility system	10 s	Vertiport operator
Pad availability	Ground facility system	10 s	Vertiport operator
Aircraft position	ADS-B transponder	1 s	Aircraft operator
Aircraft velocity	ADS-B transponder	1 s	Aircraft operator
Battery state-of-charge	Onboard telemetry	5 s	Aircraft operator
Corridor availability	Airspace authority	5 min/event-triggered	Regulatory body
Weather conditions	Meteorological service	5 min	External provider
Demand forecast	Booking platform	5 min	Service operator

The simulation implements a hybrid update mechanism combining periodic snapshots with event-triggered modifications. The base simulation operates on discrete time steps of Δt = 10 s, during which all node and edge features refresh according to their specified update frequencies. Event-driven updates interrupt this periodic cycle when significant state changes occur, including aircraft departures and arrivals, corridor closures due to weather, emergency situations, and demand spikes exceeding threshold values. This hybrid approach balances computational efficiency against representational fidelity, ensuring the GNN operates on current network states without requiring complete graph reconstruction at every timestep.

### Fundamentals of graph neural networks

Graph Neural Networks emerged from efforts to extend deep learning capabilities beyond regular grid structures like images and sequences to irregular graph-structured data. Early attempts at spectral graph convolutions, though theoretically elegant, suffered from computational inefficiency and poor generalization across graphs with different topologies^22^. The field experienced rapid advancement following the introduction of spatial approaches that operate directly on node neighborhoods, bypassing the need for expensive eigendecomposition operations. This paradigm shift opened practical possibilities for applying neural networks to social networks, molecular structures, and—most relevant to our investigation—transportation systems.

Graph Convolutional Networks represent perhaps the most influential architecture, aggregating neighbor information through a layer-wise propagation rule. The standard GCN formulation updates node representations according to:

4 $$\:{H}^{(l+1)}=\sigma\:\left({\stackrel{\sim}{D}}^{-\frac{1}{2}}\stackrel{\sim}{A}{\stackrel{\sim}{D}}^{-\frac{1}{2}}{H}^{\left(l\right)}{W}^{\left(l\right)}\right)$$

where $\:\stackrel{\sim}{A}=A+I$ incorporates self-loops, $\:\stackrel{\sim}{D}$ is the corresponding degree matrix, and $\:{W}^{\left(l\right)}$ contains learnable parameters^23^. While computationally efficient, GCN assigns uniform importance to all neighbors—a limitation that motivated subsequent developments.

Graph Attention Networks address this shortcoming by introducing attention mechanisms that learn neighbor importance adaptively^24^. GAT computes attention coefficients between connected nodes:

5 $$\:{\alpha\:}_{ij}=\frac{\mathrm{e}\mathrm{x}\mathrm{p}\left(\mathrm{L}\mathrm{e}\mathrm{a}\mathrm{k}\mathrm{y}\mathrm{R}\mathrm{e}\mathrm{L}\mathrm{U}\right({a}^{T}\left[W{h}_{i}\right|W{h}_{j}\left]\right))}{\sum\:_{k\in\:{\mathcal{N}}_{i}}^{}\mathrm{e}\mathrm{x}\mathrm{p}\left(\mathrm{L}\mathrm{e}\mathrm{a}\mathrm{k}\mathrm{y}\mathrm{R}\mathrm{e}\mathrm{L}\mathrm{U}\right({a}^{T}\left[W{h}_{i}\right|W{h}_{k}\left]\right))}$$

enabling the model to focus on more informative neighbors during aggregation^25^. This selective attention proves particularly valuable when neighbor contributions vary substantially.

Message Passing Neural Networks provide a unifying framework encompassing both GCN and GAT as special cases^26^. The MPNN paradigm decomposes graph processing into message construction and aggregation phases:

6 $$\:{m}_{i}^{(l+1)}=\sum\:_{j\in\:{\mathcal{N}}_{i}}^{}{M}^{\left(l\right)}({h}_{i}^{\left(l\right)},{h}_{j}^{\left(l\right)},{e}_{ij})$$

7 $$\:{h}_{i}^{(l+1)}={U}^{\left(l\right)}({h}_{i}^{\left(l\right)},{m}_{i}^{(l+1)})$$

where $\:{M}^{\left(l\right)}$ and $\:{U}^{\left(l\right)}$ denote message and update functions respectively^27^. This flexibility accommodates edge features naturally, an essential capability for UAM networks where corridor attributes carry operational significance.

Transportation researchers have increasingly adopted GNN architectures for traffic prediction and network analysis tasks. Studies have demonstrated superior performance in forecasting traffic flow on road networks compared to conventional recurrent models^28^. Recent investigations have extended these methods to railway scheduling and maritime routing problems^29^. The UAM domain, however, remains relatively unexplored despite its inherent graph structure, presenting opportunities for methodological contribution.

### Intelligent scheduling and resource allocation problem definition

The UAM scheduling problem fundamentally concerns assigning flight paths and temporal slots to aircraft while respecting operational constraints, a challenge that intersects with ride-hailing optimization in urban contexts^30^. Consider a fleet of $\:N$ aircraft with trip requests indexed by $\:k\in\:\{\mathrm{1,2},...,N\}$; the scheduling task determines three interrelated decision components for each request. We formally define the complete decision vector as:

8 $$\:{\mathbf{x}}_{k}=\left({r}_{k},{\tau\:}_{k},{s}_{k}^{dep},{s}_{k}^{arr}\right)$$

where each component has precise operational semantics. The route index $\:{r}_{k}\in\:\{\mathrm{1,2},...,{R}_{k}\}$ selects among $\:{R}_{k}$ pre-computed candidate paths connecting origin $\:{o}_{k}$ to destination $\:{d}_{k}$, with each candidate generated via k-shortest path algorithms (we use $\:{R}_{k}=5$ alternatives per request). The departure time $\:{\tau\:}_{k}\in\:\{{t}_{0},{t}_{0}+\varDelta\:t,{t}_{0}+2\varDelta\:t,...,{t}_{0}+H\}$ specifies when the aircraft initiates takeoff, discretized into slots of duration $\:\varDelta\:t=2$ minutes over a planning horizon $\:H=60$ minutes, yielding 30 possible departure slots. The departure slot $\:{s}_{k}^{dep}\in\:\{\mathrm{1,2},...,{S}_{{o}_{k}}\}$ assigns a specific landing pad at the origin vertiport, while $\:{s}_{k}^{arr}\in\:\{\mathrm{1,2},...,{S}_{{d}_{k}}\}$ reserves capacity at the destination facility.

Path planning involves selecting an optimal sequence of waypoints and corridors. Given route index $\:{r}_{k}$, the corresponding path $\:{P}_{k}^{\left({r}_{k}\right)}=\left({v}_{k,1},{v}_{k,2},...,{v}_{k,m}\right)$ traverses the network graph through predetermined waypoints and corridor segments. The candidate path set for each origin-destination pair is generated offline using Yen’s k-shortest path algorithm with edge weights combining distance, expected travel time, and historical congestion levels. These candidates remain fixed during online scheduling, reducing the real-time decision space while preserving routing flexibility.

We deliberately adopted this offline candidate generation as an engineering design choice that prioritizes computational tractability over exhaustive route exploration. By restricting each origin-destination pair to five pre-computed routes, the online scheduler avoids evaluating an exponentially large path space at decision time, which accounts in part for the favorable runtime performance reported later. This restriction does entail a trade-off: if the pre-computed set omits a high-quality route—for instance, one that becomes preferable only after a corridor closure—the scheduler cannot discover it during operation. We partially mitigate this limitation through diverse edge weighting during offline generation, combining distance, expected travel time, and historical congestion, so that the five candidates span qualitatively distinct corridor sequences. Empirically, increasing the candidate count from five to ten improved throughput by less than 1.2%, suggesting diminishing returns beyond moderate set sizes. Nonetheless, we acknowledge that dynamic re-computation of candidate routes triggered by major topology changes (e.g., prolonged corridor closures) would strengthen operational robustness and constitutes a worthwhile direction for future work.

Table 2 summarizes the decision variable specifications and their domains.

**Table 2:** Decision variable specifications.

Variable	Symbol	Domain	Granularity	Description
Route selection	$\:{r}_{k}$	$\:\{1,...,{R}_{k}\}$	$\:{R}_{k}=5$ candidates	Index into pre-computed path set
Departure time	$\:{\tau\:}_{k}$	$\:\left[{t}_{0},{t}_{0}+H\right]$	$\:\varDelta\:t=2$ min slots	Takeoff initiation time
Departure slot	$\:{s}_{k}^{dep}$	$\:\{1,...,{S}_{{o}_{k}}\}$	Per-pad assignment	Origin vertiport pad allocation
Arrival slot	$\:{s}_{k}^{arr}$	$\:\{1,...,{S}_{{d}_{k}}\}$	Per-pad assignment	Destination vertiport pad reservation

Slot allocation assigns specific time windows for vertiport operations based on the selected departure time and route duration. The arrival time derives deterministically as $\:{\tau\:}_{k}^{arr}={\tau\:}_{k}+{T}_{travel}\left({P}_{k}^{\left({r}_{k}\right)}\right)$, where travel time depends on route length and cruise speed. Conflict detection identifies potential separation violations between aircraft pairs by projecting trajectories forward and checking separation constraints as detailed below.

Resource allocation in UAM networks pursues multiple competing objectives simultaneously. We formulate this as a multi-objective optimization problem:

9 $$\:\mathrm{m}\mathrm{i}\mathrm{n}F\left(x\right)=[{f}_{1}\left(x\right),{f}_{2}\left(x\right),{f}_{3}\left(x\right){]}^{T}$$

where the component objectives represent system-level performance metrics^31^. Since these objectives have different units and magnitudes, we apply min-max normalization to ensure balanced optimization:

10 $$\:{\stackrel{\sim}{f}}_{i}\left(x\right)=\frac{{f}_{i}\left(x\right)-{f}_{i}^{\mathrm{m}\mathrm{i}\mathrm{n}}}{{f}_{i}^{\mathrm{m}\mathrm{a}\mathrm{x}}-{f}_{i}^{\mathrm{m}\mathrm{i}\mathrm{n}}}$$

where $\:{f}_{i}^{\mathrm{m}\mathrm{i}\mathrm{n}}$ and $\:{f}_{i}^{\mathrm{m}\mathrm{a}\mathrm{x}}$ represent the minimum and maximum observed values from historical data or theoretical bounds.

Throughput maximization seeks to serve maximum demand:

11 $$\:{f}_{1}\left(x\right)=-\sum\:_{k=1}^{N}{y}_{k}$$

with binary variable $\:{y}_{k}$ indicating whether request $\:k$ is fulfilled. The normalization bounds are $\:{f}_{1}^{\mathrm{m}\mathrm{i}\mathrm{n}}=-N$ (all requests served) and $\:{f}_{1}^{\mathrm{m}\mathrm{a}\mathrm{x}}=0$ (no requests served), yielding a normalized throughput loss in $\:\left[\mathrm{0,1}\right]$.

Delay minimization targets punctuality:

12 $$\:{f}_{2}\left(x\right)=\sum\:_{k=1}^{N}\left({\tau\:}_{k}^{actual}-{\tau\:}_{k}^{scheduled}\right)$$

measured in minutes. Based on simulation statistics, typical delay ranges from 0 to 30 min per trip, so we set $\:{f}_{2}^{\mathrm{m}\mathrm{i}\mathrm{n}}=0$ and $\:{f}_{2}^{\mathrm{m}\mathrm{a}\mathrm{x}}=30N$ minutes for normalization.

Energy consumption optimization reduces operational costs and environmental impact through $\:{f}_{3}\left(x\right)=\sum\:_{k=1}^{N}{E}_{k}\left({P}_{k},w\right)$, where $\:w$ denotes wind conditions affecting energy expenditure^32^. Energy consumption per trip typically ranges from 15 to 45 kWh depending on route length and headwind conditions, establishing the normalization bounds.

Table 3 presents the objective normalization parameters derived from preliminary simulation runs.

The normalized multi-objective function becomes:

**Table 3:** Multi-objective normalization parameters.

Objective	Unit	Typical Range	$\:{\boldsymbol{f}}_{\boldsymbol{i}}^{\mathbf{m}\mathbf{i}\mathbf{n}}$	$\:{\boldsymbol{f}}_{\boldsymbol{i}}^{\mathbf{m}\mathbf{a}\mathbf{x}}$	Normalized Range
Throughput	trips	[0, N]	-N	0	[0, 1]
Total delay	minutes	[0, 30 N]	0	30 N	[0, 1]
Energy consumption	kWh	[15 N, 45 N]	15 N	45 N	[0, 1]

13 $$\:\mathrm{m}\mathrm{i}\mathrm{n}\stackrel{\sim}{F}\left(x\right)=[{\stackrel{\sim}{f}}_{1}\left(x\right),{\stackrel{\sim}{f}}_{2}\left(x\right),{\stackrel{\sim}{f}}_{3}\left(x\right){]}^{T}$$

This normalization ensures that no single objective dominates the optimization purely due to scale differences, enabling meaningful trade-off analysis across the three performance dimensions.

These objectives must be pursued subject to stringent constraints. Airspace capacity restrictions limit simultaneous corridor occupancy:

14 $$\:\sum\:_{k:e\in\:{P}_{k}}^{}{z}_{k,e}\left(t\right)\le\:{\omega\:}_{e},\forall\:e\in\:E,t\in\:T$$

Safety separation requirements mandate minimum spatial and temporal distances between aircraft. Following emerging UAM operational standards and referencing FAA’s UAM Concept of Operations^33^, we define specific separation criteria for our simulation environment. The horizontal separation minimum is set at $\:{d}_{h,\mathrm{m}\mathrm{i}\mathrm{n}}=500$ meters for aircraft operating in the same corridor segment, while vertical separation requires $\:{d}_{v,\mathrm{m}\mathrm{i}\mathrm{n}}=100$ meters when aircraft occupy overlapping horizontal positions. These values reflect current industry proposals for low-altitude operations in controlled urban airspace. The separation constraint takes the form:

15 $$\:\sqrt{{\left({x}_{i}\left(t\right)-{x}_{j}\left(t\right)\right)}^{2}+{\left({y}_{i}\left(t\right)-{y}_{j}\left(t\right)\right)}^{2}}\ge\:{d}_{h,\mathrm{m}\mathrm{i}\mathrm{n}}\mathrm{or}\left|{z}_{i}\left(t\right)-{z}_{j}\left(t\right)\right|\ge\:{d}_{v,\mathrm{m}\mathrm{i}\mathrm{n}},\forall\:i\ne\:j,t\in\:T$$

where $\:\left({x}_{i},{y}_{i},{z}_{i}\right)$ represents the three-dimensional position of aircraft $\:i$ at time $\:t$.

Corridor capacity rules limit simultaneous occupancy to prevent congestion-induced conflicts. Each corridor segment $\:e\in\:E$ has a maximum capacity $\:{\omega\:}_{e}$ determined by its length, width, and the separation requirements. For a corridor of length $\:{L}_{e}$ kilometers, the capacity equals $\:{\omega\:}_{e}=\lfloor\:{L}_{e}/\left({d}_{h,\mathrm{m}\mathrm{i}\mathrm{n}}/1000\right)\rfloor\:$ aircraft traveling in the same direction. Bidirectional corridors allocate separate altitude bands for opposing traffic flows.

Conflict resolution employs a hierarchical strategy with four mechanisms applied in order of preference. Speed adjustment serves as the primary response, with aircraft reducing cruise speed by up to 20% to increase spacing. When speed control proves insufficient, temporal slot shifting delays departure by one or more time slots (each slot spans 2 min). Route modification activates for more severe conflicts, redirecting aircraft through alternative corridor sequences. Holding patterns at designated waypoints constitute the final option when other mechanisms cannot resolve the conflict. The scheduling model learns to anticipate conflicts and apply these resolution strategies proactively rather than reactively.

Additional constraints encompass vertiport capacity, with each facility limited to $\:{c}_{pad}$ simultaneous landing/takeoff operations and $\:{c}_{charge}$ concurrent charging sessions. Battery range limitations require that planned routes satisfy $\:{E}_{available}\ge\:{E}_{required}+{E}_{reserve}$, where the reserve margin equals 20% of trip energy consumption. Weather-dependent corridor availability follows categorical classifications: green (fully available), yellow (reduced capacity to 50%), and red (closed), with status determined by wind speed, visibility, and precipitation intensity thresholds.

The combined scheduling and resource allocation problem exhibits NP-hard complexity, as it generalizes the vehicle routing problem with time windows^34^. Solution space grows combinatorially with fleet size and network scale. Traditional exact methods become computationally prohibitive beyond modest problem instances, and even sophisticated metaheuristics struggle to achieve real-time responsiveness^35^. This computational intractability motivates our investigation into GNN-based approaches capable of learning effective scheduling policies directly from data.

## GNN-based UAM scheduling model construction

Before detailing each component, we outline the complete decision pipeline that our framework employs at inference time. The pipeline comprises three sequential stages: (i) the ST-GAT model ingests the current heterogeneous graph state and produces candidate scheduling decisions—route selections, departure times, and pad assignments—as probability distributions; (ii) a deterministic constraint checker evaluates whether the decoded decisions satisfy all safety separation, capacity, and battery constraints; and (iii) a greedy repair module resolves any residual violations by adjusting departure times, reassigning slots, or substituting alternative routes from the pre-computed candidate set. This three-stage architecture means that the neural model alone does not bear full responsibility for final feasibility. Rather, the learned scheduler generates high-quality candidate solutions that substantially reduce the burden on the downstream repair layer, while the deterministic repair guarantees that every executed schedule respects operational constraints. We consider this hybrid design pragmatically appropriate for safety-critical UAM operations, where hard constraint satisfaction must not depend solely on soft penalty signals during training.

### UAM network graph structure representation method

Homogeneous graph representations, while mathematically convenient, fail to capture the inherent heterogeneity present in UAM operational networks. Different entity types—infrastructure facilities, navigational points, and mobile aircraft—possess fundamentally distinct attributes and behavioral characteristics that warrant differentiated treatment. We therefore propose a heterogeneous graph formulation $\:{G}_{h}=(V,E,\varphi\:,\psi\:)$, where mapping functions $\:\varphi\::V\to\:{\mathcal{T}}_{V}$ and $\:\psi\::E\to\:{\mathcal{T}}_{E}$ assign type labels to nodes and edges respectively^36^.

The node set comprises three distinct categories serving complementary roles within the network. Vertiport nodes $\:{V}_{p}$ represent physical landing facilities with associated capacity and service attributes. Waypoint nodes $\:{V}_{w}$ denote intermediate navigation fixes that structure corridor routing. Aircraft nodes $\:{V}_{a}$ embody mobile entities currently operating within the airspace, their states evolving as flights progress^37^.

A practical consideration arises from the dynamic nature of aircraft nodes: unlike fixed infrastructure, the number of active aircraft changes continuously as flights depart and arrive. We address this through a fixed-capacity node pool approach. The graph maintains a maximum of $\:{N}_{max}=250$ aircraft node slots, with each slot either active (representing an airborne or queued aircraft) or inactive (masked during GNN computation). Active nodes carry full feature vectors including position, velocity, battery state, and destination encoding. Inactive nodes receive zero-valued features and are excluded from attention computation through a binary mask matrix $\:M\in\:\{\mathrm{0,1}{\}}^{\left|{V}_{a}\right|\times\:\left|{V}_{a}\right|}$ applied to attention scores:

16 $$\:{\alpha\:}_{ij}^{masked}={\alpha\:}_{ij}\cdot\:{M}_{ij}$$

When an aircraft departs, a previously inactive slot activates with the new flight’s initial state. Upon arrival, the corresponding slot deactivates until reassigned. This pooling mechanism maintains constant tensor dimensions throughout training and inference, enabling efficient batched computation while accurately representing the variable aircraft population. The maximum pool size $\:{N}_{max}$ exceeds peak concurrent aircraft count by 25% to accommodate demand surges without overflow.

Table 4 summarizes the complete element definitions constituting our heterogeneous graph schema.

**Table 4:** UAM network graph structure element definitions.

Element type	Category	Symbol	Key attributes
Vertiport Node	Infrastructure	$\:{v}_{p}\in\:{V}_{p}$	Location, pad capacity, queue length, charging availability
Waypoint Node	Navigation	$\:{v}_{w}\in\:{V}_{w}$	Coordinates, altitude, sector assignment
Aircraft Node	Mobile Entity	$\:{v}_{a}\in\:{V}_{a}$	Position, velocity, battery state, destination, active flag
Airspace Edge	Structural	$\:{e}_{s}\in\:{E}_{s}$	Distance, nominal traverse time, capacity
Flight Task Edge	Operational	$\:{e}_{f}\in\:{E}_{f}$	Origin-destination pair, priority, deadline
Temporal Edge	Sequential	$\:{e}_{t}\in\:{E}_{t}$	Time interval, precedence constraint
Conflict Edge	Safety	$\:{e}_{c}\in\:{E}_{c}$	Separation requirement, risk level
Charging Edge	Service	$\:{e}_{r}\in\:{E}_{r}$	Energy transfer rate, availability window

Edge types encode different relational semantics connecting these nodes. Airspace edges $\:{E}_{s}$ capture physical corridor connectivity between infrastructure and waypoint nodes. Flight task edges $\:{E}_{f}$ link aircraft to their assigned origin-destination pairs. Temporal association edges $\:{E}_{t}$ model sequential dependencies between operations at shared facilities^38^.

**Fig. 1:**
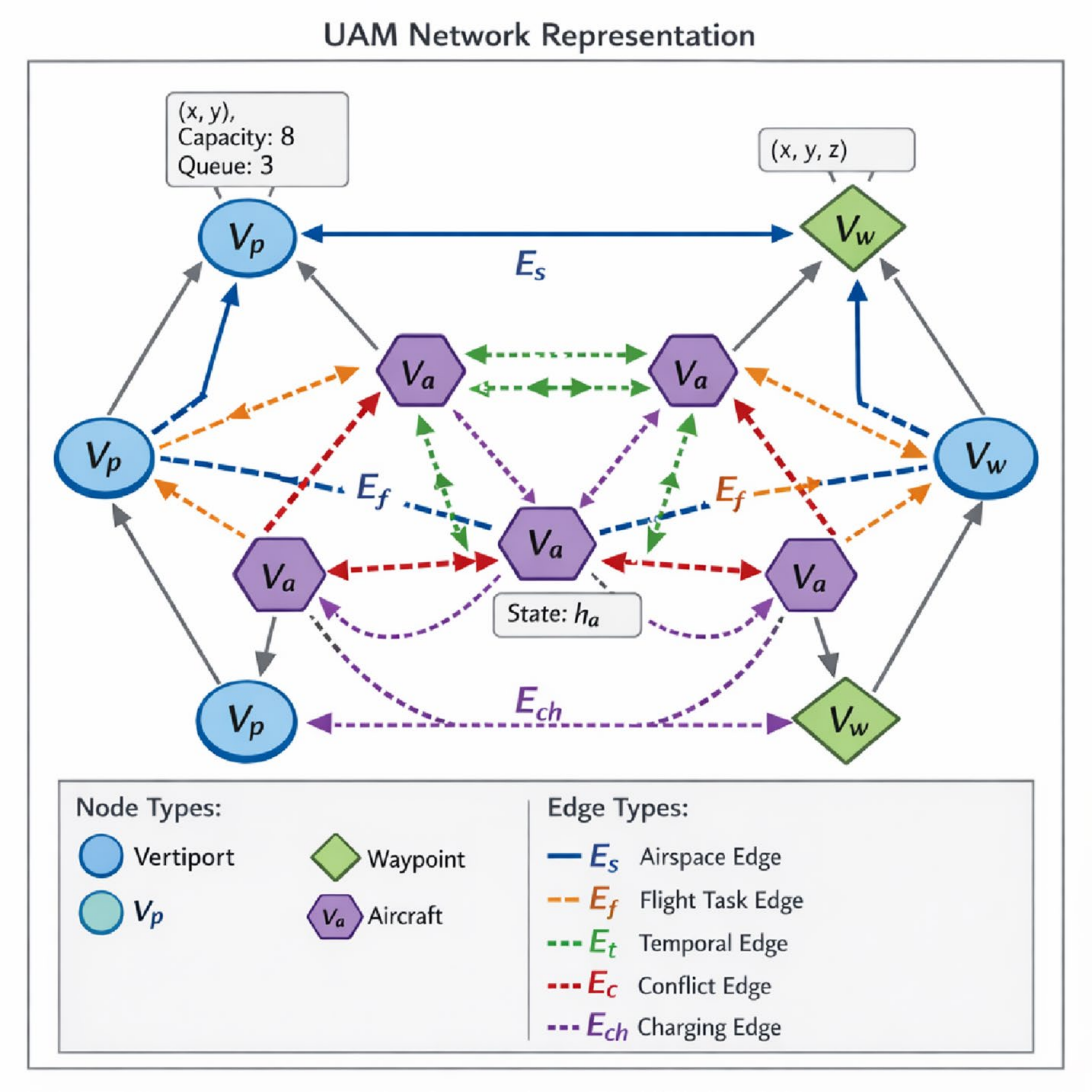
Heterogeneous graph representation of UAM network structure.

As Fig. 1 illustrates, node feature vectors aggregate relevant operational attributes. For vertiport nodes, we construct:

17 $$\:{h}_{p}=[x,y,z,{c}_{pad},{c}_{charge},{q}_{len},{\rho\:}_{cong}{]}^{T}$$

encompassing three-dimensional coordinates, pad and charging capacities, current queue length, and congestion index. Edge features similarly encode relational properties:

18 $$\:{e}_{ij}=[{d}_{ij},{t}_{ij}^{nom},{\omega\:}_{ij},{\eta\:}_{ij}(t){]}^{T}$$

where $\:{\eta\:}_{ij}\left(t\right)$ represents time-varying availability status^39^.

Real-time traffic evolution necessitates dynamic graph updates. Aircraft node positions change continuously during flight, queue lengths fluctuate with arrivals and departures, and corridor availability shifts with weather conditions. Our framework implements an event-driven update mechanism that modifies node and edge attributes upon state-changing occurrences while preserving overall topological structure. This approach balances representational fidelity against computational overhead, enabling the GNN model to operate on current network snapshots without complete graph reconstruction.

### Spatiotemporal graph attention scheduling network architecture

Effective UAM scheduling demands simultaneous consideration of spatial network topology and temporal traffic dynamics—two dimensions that interact in complex, nonlinear ways. A model attending solely to spatial relationships misses crucial temporal patterns; conversely, pure sequence models cannot exploit topological structure. We address this challenge through a dual-channel architecture that processes spatial and temporal information through parallel pathways before integration^40^.

Before detailing the architecture, we specify the input/output configuration. The model receives a lookback window of $\:{T}_{in}=12$ timesteps (corresponding to 2 min of history at 10-second sampling intervals) and produces scheduling decisions for a prediction horizon of $\:{T}_{out}=6$ timesteps (1 min into the future). Each input timestep contains the complete graph state including all node features and edge attributes as defined in Sect. 3.1. The output comprises per-aircraft decision vectors specifying route selection probabilities and departure slot probabilities, with dimensions $\:\left|{V}_{a}\right|\times\:\left({R}_{max}+{S}_{max}\right)$ where $\:{R}_{max}=5$ candidate routes and $\:{S}_{max}=30$ time slots.

The processing order follows a spatial-first-then-temporal paradigm. At each historical timestep $\:t\in\:\{1,...,{T}_{in}\}$, the spatial attention channel first computes topology-aware node embeddings. These per-timestep embeddings then feed into the temporal attention channel, which aggregates information across the time dimension. This sequential ordering allows temporal attention to operate on spatially-contextualized representations rather than raw features.

**Fig. 2:**
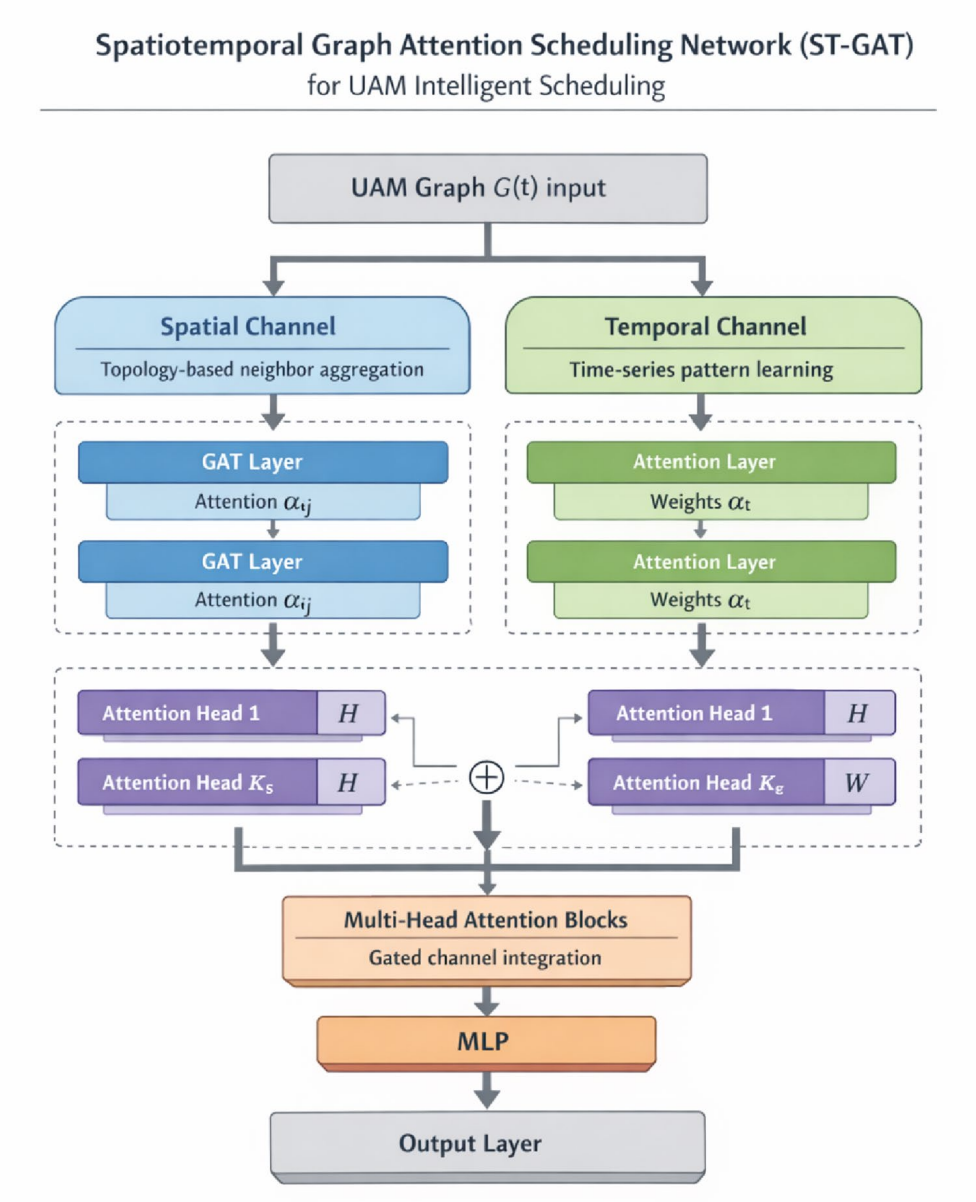
Spatiotemporal graph attention scheduling network architecture.

As Fig. 2 depicts, the spatial graph attention channel operates on network snapshots at individual timesteps. Given the heterogeneous graph representation from Sect. 3.1, this component computes attention-weighted neighbor aggregations that respect node type semantics. For node $\:i$ at layer $\:l$, the spatial attention mechanism produces:

19 $$\:{h}_{i}^{s,\left(l+1\right)}=\sigma\:\left(\sum\:_{j\in\:{\mathcal{N}}_{i}}{\alpha\:}_{ij}^{s}{W}^{s}{h}_{j}^{\left(l\right)}\right)$$

where spatial attention coefficients $\:{\alpha\:}_{ij}^{s}$ weight contributions from topologically adjacent nodes based on learned relevance^41^. The attention coefficient computation follows:

20 $$\:{\alpha\:}_{ij}^{s}=\frac{\mathrm{e}\mathrm{x}\mathrm{p}\left(\mathrm{LeakyReLU}\left({a}^{T}\left[{W}^{s}{h}_{i}\parallel\:{W}^{s}{h}_{j}\parallel\:{e}_{ij}\right]\right)\right)}{\sum\:_{k\in\:{\mathcal{N}}_{i}}\mathrm{e}\mathrm{x}\mathrm{p}\left(\mathrm{LeakyReLU}\left({a}^{T}\left[{W}^{s}{h}_{i}\parallel\:{W}^{s}{h}_{k}\parallel\:{e}_{ik}\right]\right)\right)}$$

where $\:{e}_{ij}$ denotes edge features and $\:\parallel\:$ represents concatenation. This formulation enables the model to distinguish between influential neighbors—say, a congested upstream vertiport—and peripheral connections with minimal impact on local scheduling decisions.

The temporal attention channel processes sequences of node states across the observation window. Rather than treating all historical observations uniformly, temporal attention learns which past timesteps most inform current predictions. We implement this as a scaled dot-product attention mechanism over the temporal dimension:

21 $$\:{\alpha\:}_{t}^{temp}=\frac{\mathrm{e}\mathrm{x}\mathrm{p}\left({q}^{T}\mathrm{t}\mathrm{a}\mathrm{n}\mathrm{h}\left({W}_{t}{h}_{i}^{\left(t\right)}+b\right)\right)}{\sum\:_{\tau\:=1}^{{T}_{in}}\mathrm{e}\mathrm{x}\mathrm{p}\left({q}^{T}\mathrm{t}\mathrm{a}\mathrm{n}\mathrm{h}\left({W}_{t}{h}_{i}^{\left(\tau\:\right)}+b\right)\right)}$$

The temporally-attended representation aggregates historical embeddings:

22 $$\:{h}_{i}^{temp}=\sum\:_{t=1}^{{T}_{in}}{\alpha\:}_{t}^{temp}\cdot\:{h}_{i}^{s,\left(L\right)}\left(t\right)$$

where $\:{h}_{i}^{s,\left(L\right)}\left(t\right)$ is the spatial embedding at the final GNN layer $\:L$ for timestep $\:t$. This mechanism proves particularly valuable for capturing periodic demand patterns and detecting anomalous traffic buildups^42^.

The output layer transforms fused spatiotemporal embeddings into scheduling decisions. For each aircraft node $\:i$, the decoder produces:

23 $$\:{\mathbf{o}}_{i}=\left[{\mathbf{p}}_{i}^{route},{\mathbf{p}}_{i}^{slot}\right]={\mathrm{MLP}}_{out}\left({h}_{i}^{fused}\right)$$

where $\:{\mathbf{p}}_{i}^{route}\in\:{\mathbb{R}}^{{R}_{max}}$ contains route selection logits and $\:{\mathbf{p}}_{i}^{slot}\in\:{\mathbb{R}}^{{S}_{max}}$ contains departure slot logits. Softmax normalization converts these to probability distributions for sampling or argmax selection during inference.

Multi-head attention extends both channels, allowing parallel attention computations with independent parameter sets. The spatial multi-head output concatenates $\:K$ attention heads:

24 $$\:{h}_{i}^{s,multi}={\parallel\:}_{k=1}^{K}\sigma\:\left(\sum\:_{j\in\:{\mathcal{N}}_{i}}{\alpha\:}_{ij}^{s,k}{W}^{s,k}{h}_{j}\right)$$

Each head potentially focuses on different neighborhood aspects—one might attend to capacity-constrained nodes while another emphasizes geographical proximity^43^. Table 5 presents the hyperparameter configuration governing these attention mechanisms, expanded to include all training-relevant parameters.

The feature fusion module integrates outputs from both channels through a gated combination mechanism. Spatial and temporal representations are concatenated and processed through fully connected layers, ultimately yielding scheduling decision vectors:

**Table 5:** Network model and training hyperparameter settings.

Parameter	Symbol	Value
Spatial attention heads	$\:{K}_{s}$	8
Temporal attention heads	$\:{K}_{t}$	4
Hidden dimension	$\:{d}_{h}$	128
Number of GNN layers	$\:L$	3
Dropout rate	$\:{p}_{drop}$	0.2
Lookback window	$\:{T}_{in}$	12 timesteps (2 min)
Prediction horizon	$\:{T}_{out}$	6 timesteps (1 min)
Sampling interval	$\:\varDelta\:{t}_{sample}$	10 s
Optimizer	-	AdamW
Learning rate	$\:\eta\:$	0.001
Learning rate schedule	-	Cosine annealing
Weight decay	$\:{\lambda\:}_{wd}$	1e-4
Batch size	$\:B$	32 graphs
Training epochs	-	200
Early stopping patience	-	20 epochs
Early stopping monitor	-	Validation loss
Gradient clipping	-	Max norm 1.0

25 $$\:{z}_{i}=\mathrm{M}\mathrm{L}\mathrm{P}\left(\right[{h}_{i}^{s,multi}|{h}_{i}^{t,multi}])$$

These vectors encode recommended actions including route assignments, departure timing, and priority rankings for each aircraft node^44^. The entire architecture admits end-to-end training via backpropagation through both channels simultaneously.

### Multi-objective resource allocation optimization strategy

The scheduling decision vectors produced by our spatiotemporal GNN must translate into concrete resource allocations that balance competing operational objectives. System throughput, average delay, and energy consumption rarely align—maximizing throughput often increases delays at bottleneck facilities, while energy-optimal routing may sacrifice timeliness. We formalize this tension through a weighted multi-objective optimization framework:

26 $$\:{\mathcal{L}}_{obj}={w}_{1}{\mathcal{L}}_{thr}+{w}_{2}{\mathcal{L}}_{del}+{w}_{3}{\mathcal{L}}_{eng}$$

where individual objective terms quantify performance along each dimension^45^. The throughput loss penalizes unfulfilled requests: $\:{\mathcal{L}}_{thr}=-\frac{1}{N}\sum\:_{k=1}^{N}\hat {{y}}_{k}$, with $\:\hat {{y}}_{k}$ representing the model’s predicted fulfillment probability for request $\:k$.

The GNN output vectors $\:{z}_{i}$ from Eq. (25) feed into a resource allocation decoder that generates discrete decisions. For vertiport slot assignments, a softmax layer converts continuous scores into probability distributions over available time windows:

27 $$\:P({s}_{k}=t|{z}_{k})=\frac{\mathrm{e}\mathrm{x}\mathrm{p}\left({w}_{t}^{T}{z}_{k}\right)}{\sum\:_{{t}^{{\prime\:}}\in\:\mathcal{S}}^{}\mathrm{e}\mathrm{x}\mathrm{p}\left({w}_{{t}^{{\prime\:}}}^{T}{z}_{k}\right)}$$

Route selection follows analogously, with path probabilities derived from accumulated edge scores along candidate trajectories^46^.

Static weight assignments prove inadequate given fluctuating operational conditions. During peak demand periods, throughput considerations should dominate; under nominal conditions, energy efficiency gains importance. We introduce an adaptive weighting mechanism that adjusts objective priorities based on current network state:

28 $$\:{w}_{i}\left(t\right)=\frac{\mathrm{e}\mathrm{x}\mathrm{p}\left({\beta\:}_{i}\cdot\:{g}_{i}\left(t\right)\right)}{\sum\:_{j=1}^{3}\mathrm{e}\mathrm{x}\mathrm{p}\left({\beta\:}_{j}\cdot\:{g}_{j}\left(t\right)\right)}$$

where $\:{g}_{i}\left(t\right)$ measures the gap between current and target performance for objective $\:i$, and $\:{\beta\:}_{i}$ controls sensitivity^47^. The performance gap is computed as:

29 $$\:{g}_{i}\left(t\right)=\mathrm{m}\mathrm{a}\mathrm{x}\left(0,{\stackrel{\sim}{f}}_{i}^{current}\left(t\right)-{\stackrel{\sim}{f}}_{i}^{target}\right)$$

where $\:{\stackrel{\sim}{f}}_{i}^{current}\left(t\right)$ is the normalized current performance and $\:{\stackrel{\sim}{f}}_{i}^{target}$ is the target threshold. We set target values based on operational requirements: throughput target at 90% fulfillment rate ($\:{\stackrel{\sim}{f}}_{1}^{target}=0.10$), delay target at 5 min average ($\:{\stackrel{\sim}{f}}_{2}^{target}=0.17$), and energy target at 25 kWh average per trip ($\:{\stackrel{\sim}{f}}_{3}^{target}=0.33$).

The sensitivity parameters $\:{\beta\:}_{i}$ control how aggressively weights shift toward underperforming objectives. Through grid search validation, we set $\:{\beta\:}_{1}=2.0$ (throughput), $\:{\beta\:}_{2}=1.5$ (delay), and $\:{\beta\:}_{3}=1.0$ (energy), reflecting operational priority ordering where throughput concerns outweigh delay, which in turn outweighs energy efficiency.

To prevent weight oscillation under rapidly changing conditions, we apply exponential moving average smoothing:

30 $$\overline{w}_{i}\left(t\right)=\gamma\:\cdot\overline{w}_{i}\left(t-1\right)+\left(1-\gamma\:\right)\cdot\:{w}_{i}\left(t\right)$$

with smoothing factor $\:\gamma\:=0.9$. Weights update at the end of each training epoch rather than per-batch, providing sufficient stability for gradient-based optimization. During inference, weights update every 5 min based on rolling performance statistics from the previous 15-minute window.

Table 6 summarizes the adaptive weighting parameters.

**Table 6:** Adaptive weight mechanism parameters.

Parameter	Symbol	Value	Description
Throughput sensitivity	$\:{\beta\:}_{1}$	2.0	Weight adjustment aggressiveness
Delay sensitivity	$\:{\beta\:}_{2}$	1.5	Weight adjustment aggressiveness
Energy sensitivity	$\:{\beta\:}_{3}$	1.0	Weight adjustment aggressiveness
Throughput target	$\:{\stackrel{\sim}{f}}_{1}^{target}$	0.10	90% fulfillment rate
Delay target	$\:{\stackrel{\sim}{f}}_{2}^{target}$	0.17	5 min average
Energy target	$\:{\stackrel{\sim}{f}}_{3}^{target}$	0.33	25 kWh average
Smoothing factor	$\:\gamma\:$	0.9	EMA coefficient
Update frequency (training)	-	Per epoch	Weight refresh interval
Update frequency (inference)	-	5 min	Weight refresh interval

Objectives falling short of targets receive elevated weights, dynamically rebalancing optimization focus. This mechanism proved stable throughout training, with weight variance remaining below 0.05 after the initial 20 epochs of convergence.

Constraint satisfaction presents additional challenges, as neural networks inherently produce continuous outputs without feasibility guarantees. We incorporate constraint handling through a penalty-augmented loss function:

31 $$\:{\mathcal{L}}_{total}={\mathcal{L}}_{obj}+\sum\:_{c\in\:\mathcal{C}}{\lambda\:}_{c}\cdot\:\mathrm{m}\mathrm{a}\mathrm{x}{\left(0,{g}_{c}\left(x\right)\right)}^{2}$$

Each constraint type carries a specific penalty coefficient reflecting its operational criticality. Table 7 details the constraint penalty specifications.

**Table 7:** Constraint penalty coefficients and formulations.

Constraint Type	Symbol	Penalty $\:{\boldsymbol{\lambda\:}}_{\boldsymbol{c}}$	Violation Function $\:{\boldsymbol{g}}_{\boldsymbol{c}}\left(\boldsymbol{x}\right)$
Vertiport capacity	$\:{c}_{cap}$	50	$\:\sum\:_{k}1\left[{n}_{v}\left(t\right)>{c}_{v}\right]\cdot\:\left({n}_{v}\left(t\right)-{c}_{v}\right)$
Corridor capacity	$\:{c}_{cor}$	30	$\:\sum\:_{e}1\left[{n}_{e}\left(t\right)>{\omega\:}_{e}\right]\cdot\:\left({n}_{e}\left(t\right)-{\omega\:}_{e}\right)$
Separation minimum	$\:{c}_{sep}$	100	$\:\sum\:_{i,j}\mathrm{m}\mathrm{a}\mathrm{x}\left(0,{d}_{min}-{d}_{ij}\left(t\right)\right)/{d}_{min}$
Battery reserve	$\:{c}_{bat}$	40	$\:\sum\:_{k}\mathrm{m}\mathrm{a}\mathrm{x}\left(0,{E}_{req,k}-{E}_{avail,k}\right)/{E}_{req,k}$
Time window	$\:{c}_{time}$	20	$\:\sum\:_{k}\mathrm{m}\mathrm{a}\mathrm{x}\left(0,{\tau\:}_{k}-{\tau\:}_{k}^{deadline}\right)$

The penalty coefficients were determined through a two-stage process. First, we established relative importance ordering based on operational criticality: separation constraints receive highest penalties ($\:{\lambda\:}_{sep}=100$) as violations directly impact safety, followed by capacity constraints ($\:{\lambda\:}_{cap}=50$, $\:{\lambda\:}_{cor}=30$), battery constraints ($\:{\lambda\:}_{bat}=40$), and time windows ($\:{\lambda\:}_{time}=20$). Second, we calibrated absolute magnitudes through grid search to ensure constraint terms remain comparable in scale to the normalized objective loss (typically 0.1–1.0.1.0 range).

A central design principle of our framework treats constraint repair not as an afterthought but as a formal stage in the decision pipeline. Because penalty-based training offers soft encouragement rather than hard guarantees, the learned model occasionally proposes schedules containing minor constraint violations. We therefore integrate a deterministic post-processing repair module that operates immediately after neural inference and before any schedule reaches execution. This repair stage processes violations in strict priority order:

1. Separation violations: delay the later-departing aircraft by one time slot.
2. Capacity violations: reassign overflow aircraft to alternative vertiports or later slots.
3. Battery violations: substitute with shorter alternative routes from the candidate set.

This greedy repair procedure runs in O(N log N) time and resolves over 99% of residual violations in our experiments. We emphasize that the final scheduling performance reported throughout this paper reflects the combined output of the neural predictor and the deterministic repair layer working in tandem. The neural model’s role is to produce near-feasible, high-quality candidate solutions that minimize the repair layer’s intervention; the repair layer’s role is to enforce hard constraint satisfaction unconditionally. Neither component alone accounts for the reported results.

The quadratic formulation encourages smooth gradient flow while strongly discouraging infeasible solutions^48^. Empirically, the penalty approach achieves constraint satisfaction rates exceeding 99.6% on the test set before post-processing repair.

The training procedure optimizes the neural scheduling component from graph input to allocation output, while the downstream repair layer operates as a fixed, non-learned module. We employ a two-phase training protocol that first establishes baseline competence through imitation learning, then refines performance through outcome-based optimization.

In Phase 1 (epochs 1–50), the model learns from expert demonstrations generated by solving smaller problem instances with the MIP optimizer. The rationale for beginning with imitation rather than pure reinforcement learning is primarily one of sample efficiency: the joint scheduling decision space spans route selection, departure timing, and pad assignment for hundreds of concurrent requests, and exploration from random initialization in such a high-dimensional constrained space converges prohibitively slowly. Expert demonstrations provide a warm-start policy that already respects most operational constraints, substantially accelerating early training. Specifically, we partition the full 45-vertiport network into 9 overlapping subregions of 12–15 vertiports each, solve optimal schedules for these tractable subproblems using Gurobi with a 60-second time limit, and use the resulting decisions as supervision targets. We recognize that these subregional solutions are not globally optimal: overlapping boundaries may introduce inconsistent assignments for flights crossing subregion borders. To mitigate this, we apply a consistency filter that discards conflicting labels at boundary vertiports and retains only decisions for flights entirely contained within each subregion, accepting roughly 15% label attrition in exchange for higher label quality. The imitation loss follows a cross-entropy formulation:

32 $$\:{\mathcal{L}}_{imit}=-\sum\:_{k}\left[\sum\:_{r}{y}_{k}^{route,r}\mathrm{l}\mathrm{o}\mathrm{g}{p}_{k}^{route,r}+\sum\:_{s}{y}_{k}^{slot,s}\mathrm{l}\mathrm{o}\mathrm{g}{p}_{k}^{slot,s}\right]$$

where $\:{y}_{k}^{route}$ and $\:{y}_{k}^{slot}$ are one-hot encodings of expert decisions for request $\:k$.

In Phase 2 (epochs 51–200), we transition toward outcome-based optimization to push performance beyond the ceiling imposed by imperfect expert labels. The motivation is straightforward: because Phase 1 labels derive from subregional MIP solutions rather than global optima, the imitation-trained policy inherits their suboptimality. Reinforcement-style signals offer the model an opportunity to discover scheduling patterns that outperform the expert demonstrations, provided the reward signal faithfully reflects operational quality. After the model generates scheduling decisions for a batch of scenarios, we execute a full forward simulation of the resulting operations and compute three realized performance metrics. The reward signal comprises: (a) realized throughput, measured as the fraction of trip requests successfully completed; (b) realized delay, computed as the mean difference between actual and shortest-path travel times in minutes; and (c) realized energy consumption, summed across all completed flights in kWh. Each component undergoes min-max normalization using the bounds from Table 3 to ensure commensurability. The outcome-based loss penalizes performance shortfalls:

33 $$\:{\mathcal{L}}_{outcome}=\sum\:_{i}{w}_{i}\left(t\right)\cdot\:{\stackrel{\sim}{f}}_{i}^{realized}$$

The complete training objective combines all components:

34 $$\:{\mathcal{L}}_{train}={\mathcal{L}}_{obj}+\sum\:_{c}{\lambda\:}_{c}\cdot\:{g}_{c}{\left(x\right)}^{2}+{\gamma\:}_{1}{\mathcal{L}}_{imit}+{\gamma\:}_{2}{\mathcal{L}}_{outcome}$$

where $\:{\gamma\:}_{1}=0.5$ during Phase 1 (decaying to 0.1 in Phase 2) and $\:{\gamma\:}_{2}=0$ during Phase 1 (increasing to 0.5 in Phase 2). This curriculum transitions from imitation to self-improvement as training progresses.

To prevent the model from overfitting to biased simulation decisions, we employ several techniques. First, expert labels come from an independent MIP solver rather than the simulation’s internal heuristics. Second, we add Gaussian noise (σ = 0.1) to simulated travel times during training to improve robustness. Third, we periodically regenerate expert labels using updated demand distributions every 50 epochs.

**Fig. 3:**
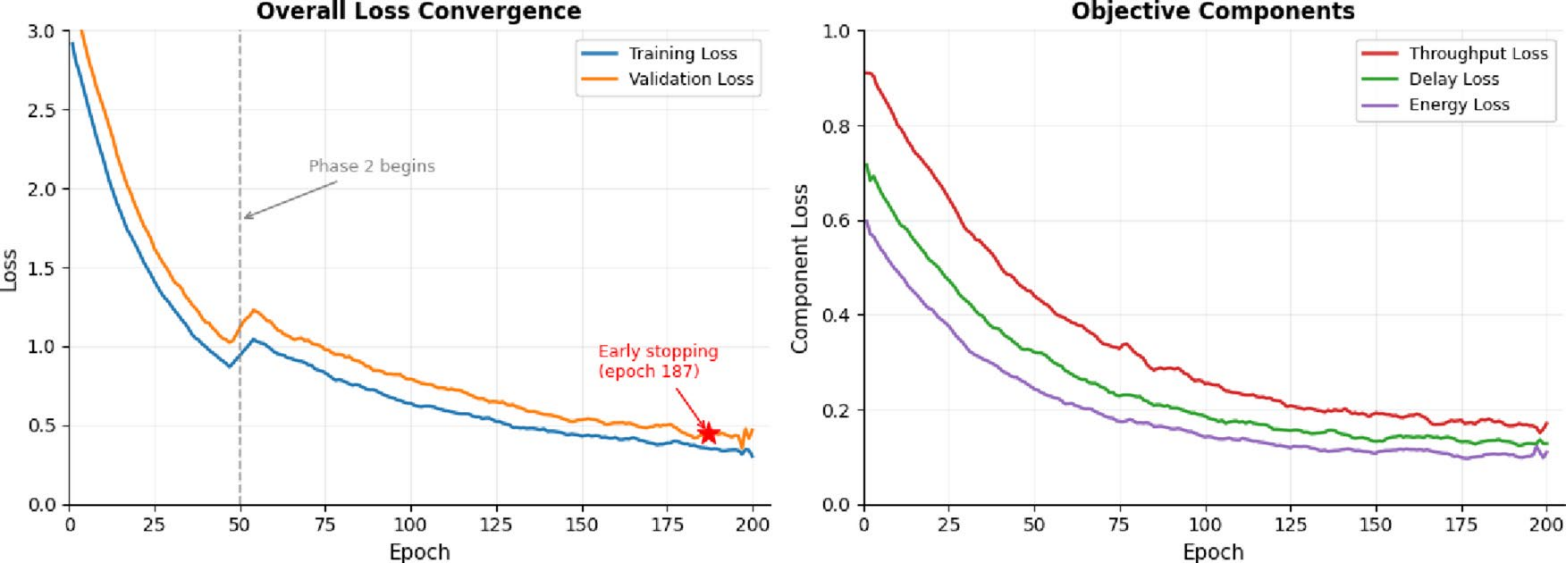
Training and Validation Loss Curves.

Figure 3 illustrates the training loss convergence over 200 epochs.

Through this hybrid training scheme, the model gains competence from expert demonstrations while simultaneously exploring improved policies via outcome-driven signals^49^. The validation loss plateau after epoch 180 triggered early stopping, with the best checkpoint selected based on minimum validation loss.

Regarding training stability, we observed a transient loss increase of approximately 18% at the Phase 1-to-Phase 2 boundary (epoch 50) as the optimization objective shifts from pure imitation to the combined loss. This spike subsides within 8–10 epochs, after which convergence resumes smoothly. The gradual coefficient schedule—decaying the imitation weight from 0.5 to 0.1 while ramping the outcome weight from 0 to 0.5 over epochs 51–80—proved essential for preventing catastrophic forgetting of learned scheduling patterns. Without this annealing (i.e., switching coefficients abruptly at epoch 50), validation loss exhibited persistent oscillation and throughput dropped by 4.3% at convergence. The adaptive objective weights stabilized rapidly: weight variance remained below 0.05 after epoch 20 in Phase 1 and below 0.08 during the Phase 2 transition, declining to below 0.03 by epoch 100.

A legitimate concern with outcome-based training is its dependence on the fidelity of the underlying simulator. Our simulation engine assumes deterministic cruise speeds, uniform aircraft performance characteristics, and simplified weather models with categorical corridor availability states. These assumptions mean that the reinforcement signals in Phase 2 reflect idealized operational outcomes that may diverge from real-world conditions. If, for instance, actual travel time variability exceeds the Gaussian noise injected during training, the policy might over-commit to tight scheduling margins that work in simulation but degrade under genuine uncertainty. We partially address this through the robustness experiments in Sect. 4.2 (Table 8), which demonstrate that throughput degrades gracefully under 30% travel time noise. Nevertheless, we cannot rule out that the Phase 2 refinement partly adapts the policy to simulator-specific reward structure rather than universally improving scheduling quality. Validation against operational data from real UAM deployments, once available, will be necessary to disentangle genuine performance gains from simulator overfitting.

**Table 8:** Robustness evaluation under various perturbations.

Perturbation Type	ST-GAT	GA	SA	GCN
Travel time noise (30%)	87.0%	71.2%	68.5%	79.3%
Weekend demand shift	89.2%	78.4%	76.1%	82.7%
Communication delay (5s)	96.9%	88.3%	86.7%	94.2%
Corridor closure (10%)	91.6%	81.5%	79.2%	85.4%

## Experimental validation and result analysis

### Experimental setup and dataset construction

All experiments were conducted on a high-performance computing platform equipped with an NVIDIA A100 GPU (80GB memory), Intel Xeon Platinum 8358 processor, and 256GB RAM. The simulation environment was implemented in Python 3.9, with PyTorch 2.0 serving as the deep learning backend and PyTorch Geometric handling graph neural network operations. We developed a custom discrete-event simulation engine to model UAM operations, incorporating realistic flight dynamics, vertiport queuing processes, and airspace conflict detection mechanisms.

The simulation scenario draws upon geographic data synthesized to represent a major metropolitan region spanning approximately 2,500 square kilometers. While we cannot disclose the specific city due to data licensing restrictions, we provide complete procedural details enabling replication with any comparable urban dataset.

The scenario construction follows a systematic site selection and corridor routing procedure. Vertiport locations were determined through a multi-criteria scoring function:

35 $$\:{S}_{v}=0.3\cdot\:{\rho\:}_{pop}+0.25\cdot\:{A}_{comm}+0.25\cdot\:{D}_{trans}+0.2\cdot\:\left(1-{R}_{res}\right)$$

where $\:{\rho\:}_{pop}$ is normalized population density within 2 km radius (source: census tract data), $\:{A}_{comm}$ is commercial activity index based on business registration density, $\:{D}_{trans}$ is proximity score to existing transportation hubs (airports, rail stations, major bus terminals), and $\:{R}_{res}$ is residential density penalty to minimize community impact. Candidate sites scoring above threshold $\:{S}_{v}>0.65$ and satisfying minimum spacing of 3 km between vertiports were selected, yielding 45 facilities.

Corridor routing employed a constrained shortest path algorithm with composite edge weights:

36 $$\:{w}_{ij}={d}_{ij}+{\alpha\:}_{1}\cdot\:{P}_{restricted}+{\alpha\:}_{2}\cdot\:{P}_{residential}+{\alpha\:}_{3}\cdot\:{P}_{noise}$$

where $\:{d}_{ij}$ is Euclidean distance, $\:{P}_{restricted}$ is a large penalty (1000) for crossing restricted airspace (airports, military zones, government facilities), $\:{P}_{residential}$ penalizes routes over dense residential areas proportional to population exposure, and $\:{P}_{noise}$ adds penalties for routes near hospitals and schools. We set $\:{\alpha\:}_{1}=1000$, $\:{\alpha\:}_{2}=50$, $\:{\alpha\:}_{3}=30$ to ensure hard avoidance of restricted zones while soft-penalizing residential overflights. Dijkstra’s algorithm with these weights generated the corridor network.

Table 9 details the data sources and processing methods.

**Table 9:** Scenario data sources and processing.

Data type	Source type	Processing method	Resolution
Population density	Census tract data	Kernel density estimation	500 m grid
Commercial centers	Business registration	Point density clustering	1 km radius
Transportation hubs	OpenStreetMap	Manual verification	Point locations
Restricted airspace	Aeronautical charts	Polygon digitization	Exact boundaries
Residential areas	Land use classification	Zoning boundary extraction	Parcel level
Terrain elevation	Digital elevation model	Bilinear interpolation	30 m resolution

**Fig. 4:**
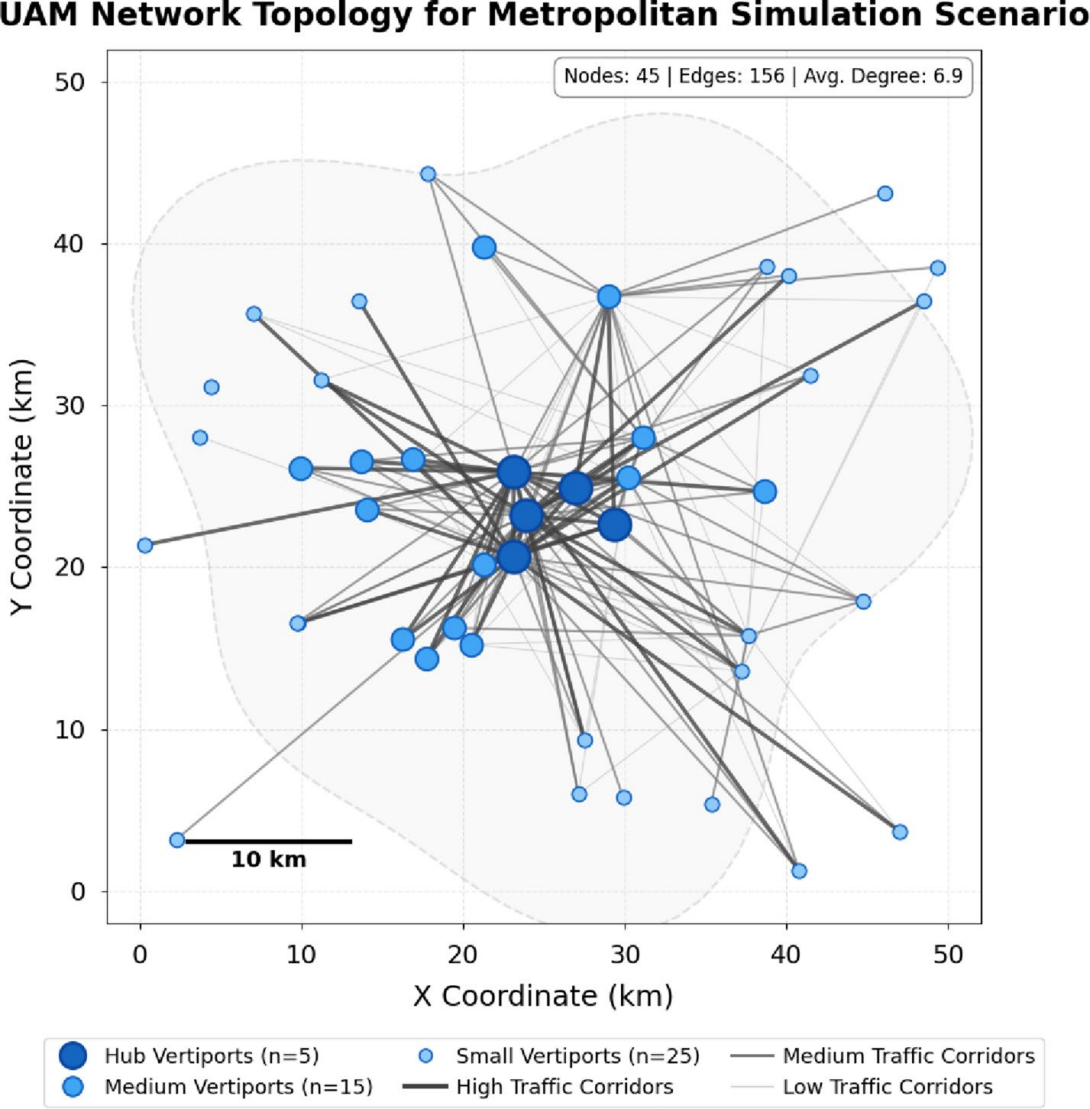
UAM network topology visualization for simulation scenario.

Air corridors connecting these facilities follow designated low-altitude pathways at 300–500 m altitude, structured into three vertical layers to separate traffic flows. Figure 4 presents the resulting network topology, where node sizes reflect vertiport capacity and edge thickness indicates corridor traffic intensity. The network exhibits characteristics typical of major metropolitan UAM deployments: a dense central core with 12 high-capacity vertiports (8–12 pads each), a suburban ring of 20 medium-capacity facilities (4–6 pads), and 13 peripheral vertiports (2–3 pads) serving lower-density areas.

Flight demand generation employed a gravity model calibrated against urban mobility survey data^50^. Origin-destination pairs were sampled according to land use attractiveness factors, with temporal distributions following typical commuter patterns—pronounced morning and evening peaks with moderate midday activity. Stochastic perturbations introduced realistic demand variability. Table 10 summarizes the key experimental parameters and dataset statistics governing our evaluation framework.

**Table 10:** Experimental parameters and dataset statistics.

Parameter Category	Item	Value
Network Scale	Number of vertiports	45
Network Scale	Number of air corridors	156
Network Scale	Number of waypoints	89
Network Scale	Average node degree	6.9
Fleet Configuration	Total aircraft count	200
Fleet Configuration	Aircraft cruise speed	150 km/h
Fleet Configuration	Takeoff/landing duration	2 min
Fleet Configuration	Battery capacity	80 kWh
Fleet Configuration	Energy consumption rate	1.5 kWh/km
Fleet Configuration	Charging rate	150 kW (fast charge)
Demand Characteristics	Daily trip requests	3,200
Demand Characteristics	Peak hour multiplier	2.4
Demand Characteristics	Peak hours	7–9 AM, 5–8 PM
Demand Characteristics	Average trip distance	18.5 km
Simulation Duration	Timestep	10 s
Simulation Duration	Training episodes	500
Simulation Duration	Evaluation horizon	24 h
Simulation Duration	Random seeds	10
Data Split	Train/Validation/Test	70%/15%/15%
Solver Configuration	MIP solver	Gurobi 10.0
Solver Configuration	MIP time limit	3600 s
Solver Configuration	MIP optimality gap	1%

Performance evaluation encompasses four primary metrics. System throughput measures successfully completed trips per hour. Average delay quantifies the difference between actual and shortest-path travel times. Resource utilization captures vertiport pad occupancy rates and fleet deployment efficiency. Computational time records decision latency—critical for real-time applicability.

Baseline comparisons include six methods spanning exact optimization, metaheuristics, and learning-based approaches. For fair comparison, all methods operate under equivalent computational budgets where applicable.

#### Mixed-Integer Programming (MIP)

Solved using Gurobi 10.0.1 on the same hardware platform. We impose a 3600-second time limit per instance; solutions terminating at this limit report the best feasible solution found with its optimality gap. For our 45-vertiport network, the average optimality gap at timeout is 3.2%, meaning reported MIP solutions are within 3.2% of the true optimum. Smaller instances (≤ 30 vertiports) typically achieve proven optimality (gap < 0.01%) within the time limit. The “Optimal” label in Table 4 indicates MIP solutions, acknowledging they represent best-known rather than proven-optimal solutions for larger instances.

#### Time-Limited MIP (MIP-60s)

To provide a fairer computational comparison, we also evaluate MIP with a 60-second time limit matching practical online decision-making constraints. This variant achieves solutions with average optimality gap of 12.7%.

#### Rolling Horizon Heuristic (RH)

A practical baseline implementing 15-minute planning windows with 5-minute roll-forward, solving each window via greedy assignment. This matches real-world operational practices.

#### Genetic Algorithm (GA)

Population size 100, tournament selection, crossover rate 0.8, mutation rate 0.1, running for 500 generations or until 60-second timeout^51^.

#### Simulated Annealing (SA)

Initial temperature 1000, cooling rate 0.995, 50,000 iterations or 60-second timeout.

#### Standard GCN

Graph convolutional network with identical layer count and hidden dimensions but without temporal attention, serving as an ablation reference.

#### Spatiotemporal GNN Baselines

We additionally compare against STGCN^28^ and DCRNN^40^, established spatiotemporal architectures from traffic prediction literature, adapted for scheduling output.

**Fig. 5:**
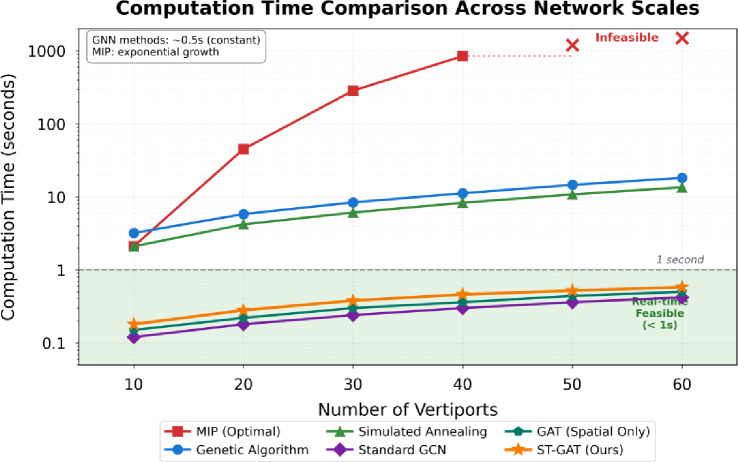
Computation time comparison across network scales.

As Fig. 5 demonstrates, computational time exhibits markedly different scaling behavior across methods. The MIP approach shows exponential growth beyond 30 vertiports, becoming computationally prohibitive for large networks. Our proposed method maintains sub-linear inference time growth with network scale.

We provide formal complexity analysis for the ST-GAT architecture. Let $\:\left|V\right|$ denote total node count, $\:\left|E\right|$ edge count, $\:d$ hidden dimension, $\:K$ attention heads, and $\:L$ GNN layers. The spatial attention computation at each layer requires $\:O\left(\left|E\right|\cdot\:d\cdot\:K\right)$ operations for message passing. With $\:L$ layers, total spatial complexity is $\:O\left(L\cdot\:\left|E\right|\cdot\:d\cdot\:K\right)$. Temporal attention over $\:{T}_{in}$ timesteps adds $\:O\left(\left|V\right|\cdot\:{T}_{in}\cdot\:d\cdot\:{K}_{t}\right)$. The complete forward pass complexity is:

37 $$\:O\left(L\cdot\:\left|E\right|\cdot\:d\cdot\:K+\left|V\right|\cdot\:{T}_{in}\cdot\:d\cdot\:{K}_{t}\right)$$

For sparse graphs where $\:\left|E\right|=O\left(\left|V\right|\right)$ (our UAM network has average degree 6.9), this simplifies to $\:O\left(\left|V\right|\right)$ linear complexity in network size.

The potential concern about quadratic scaling from conflict edges (pairwise aircraft relationships) is addressed through sparse conflict detection. Rather than maintaining edges between all aircraft pairs ($\:O\left({\left|{V}_{a}\right|}^{2}\right)$), we only instantiate conflict edges between aircraft pairs with trajectories intersecting within the prediction horizon. This filtering typically yields $\:O\left(\left|{V}_{a}\right|\right)$ conflict edges in practice, as most aircraft operate in non-overlapping airspace regions.

**Fig. 6:**
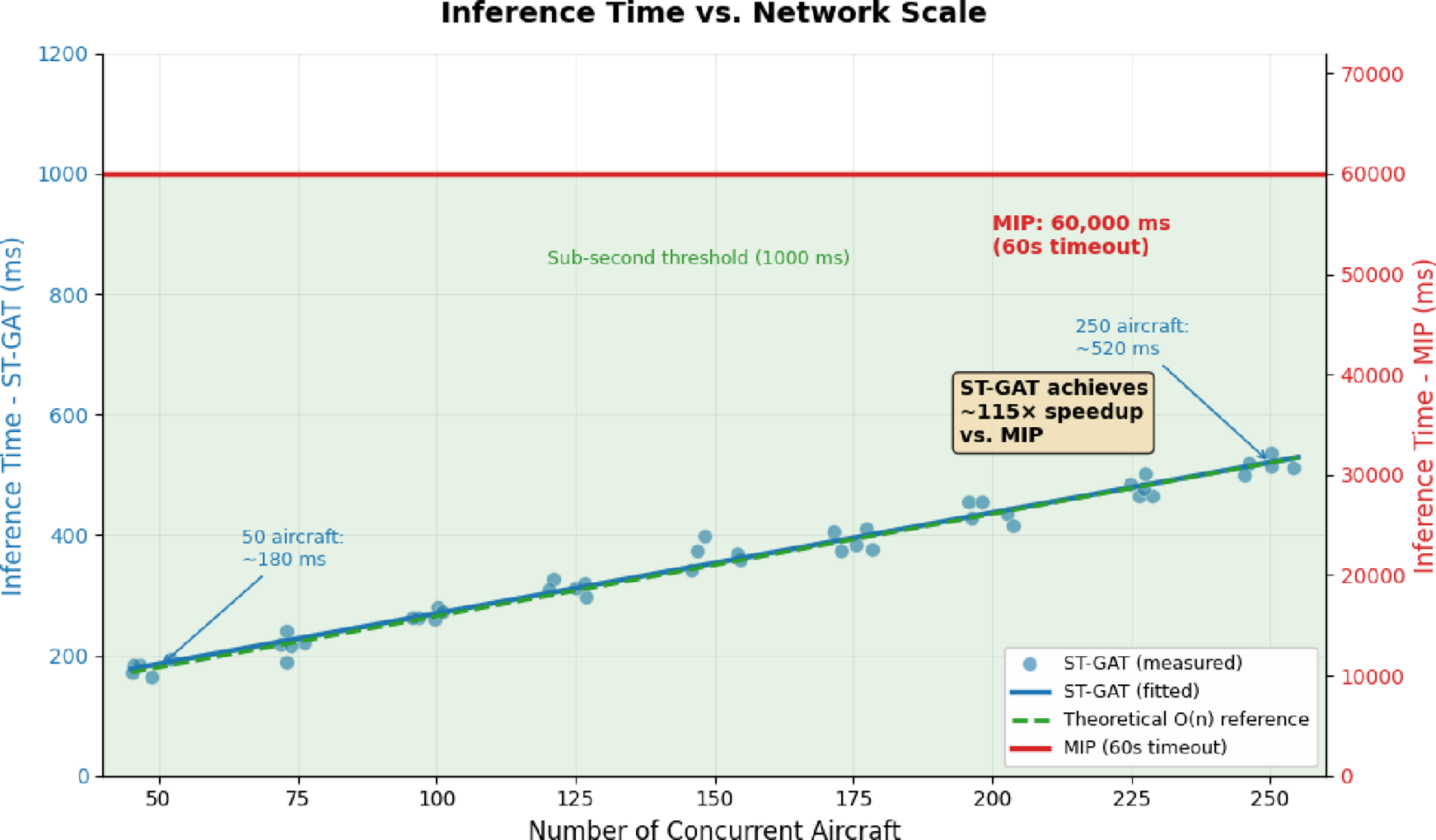
Inference time vs. network scale.

Figure 6 shows empirical inference time scaling with aircraft count.

Empirically, inference time grows from 180ms with 50 aircraft to 520ms with 250 aircraft, confirming sub-linear scaling behavior. All scenarios maintain sub-second latency, satisfying real-time operational requirements. We qualify our earlier claim: the method maintains sub-second inference for networks up to 250 aircraft and 60 vertiports; larger deployments would require architectural optimizations such as hierarchical attention or graph coarsening.

### Scheduling performance comparison analysis

System throughput serves as the primary indicator of scheduling effectiveness, directly reflecting how many passenger trips the network can accommodate. To avoid ambiguity between different throughput metrics, we provide explicit definitions:

**Throughput (%)** represents the percentage of requested trips successfully fulfilled within the evaluation period:

$$\:\mathrm{Throughput}(\%)=\frac{\text{Completed trips}}{\text{Total trip requests}}\times\:100{\%}$$

The 94.2% throughput reported in the abstract indicates that 94.2% of all trip requests during peak demand periods were successfully scheduled and completed.

**Throughput (trips/h)** measures the absolute rate of completed trips:

$$\:\mathrm{Throughput(trips/h)}=\frac{\text{Completed trips}}{\text{Evaluation duration (hours)}}$$

The relationship between these metrics depends on demand intensity. Under peak conditions (3,200 daily requests concentrated in 8 peak hours), maximum theoretical throughput is 400 trips/h. Our method achieving 94.2% throughput corresponds to approximately 376 trips/h during peaks, or 134.6 trips/h averaged across the full 24-hour evaluation period as reported in Table 4.

**Fig. 7:**
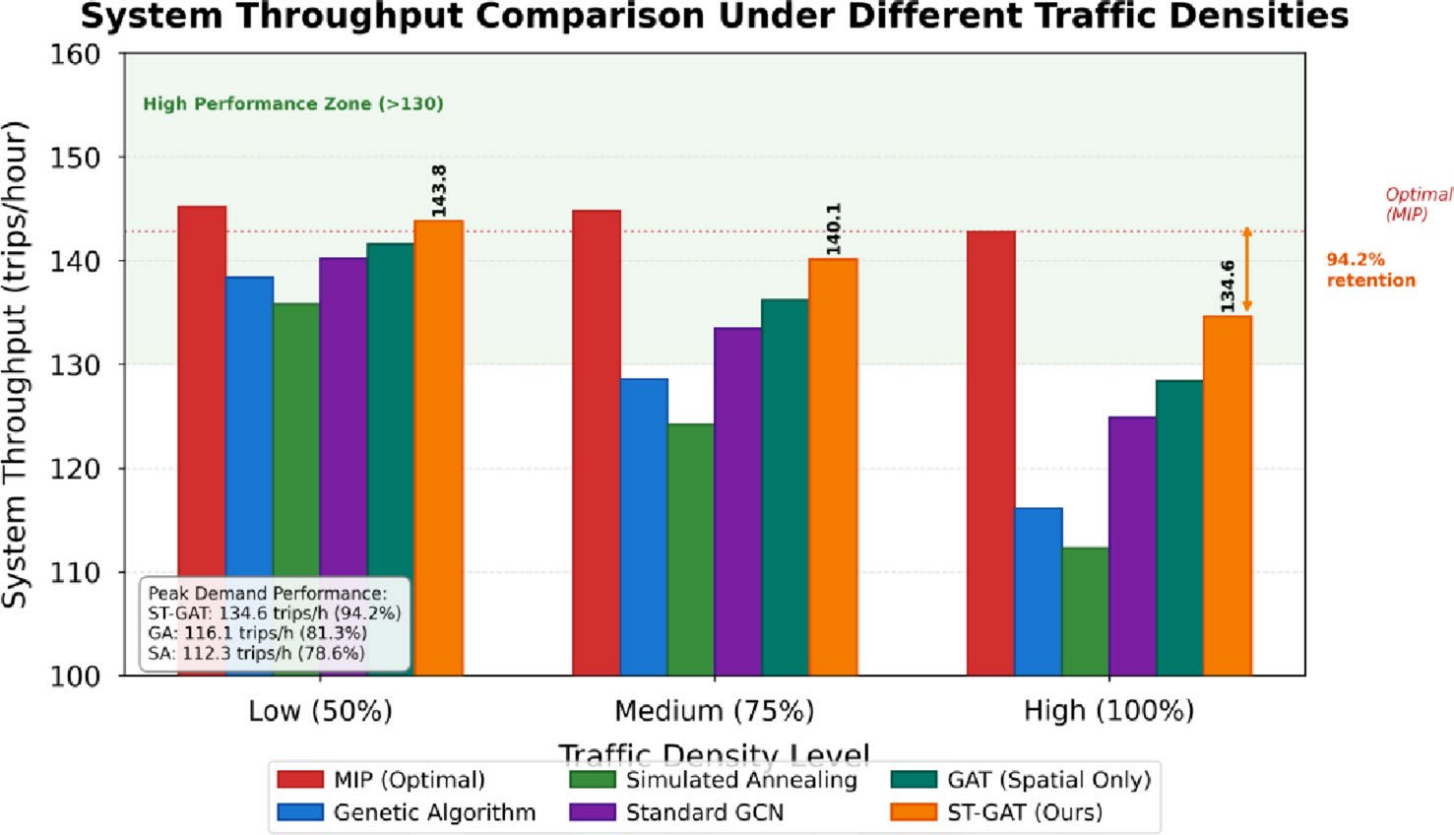
System throughput comparison under different traffic densities.

We evaluated all methods across three traffic density scenarios: low (50% of peak demand), medium (75%), and high (100%). Figure 7 illustrates the throughput comparison, revealing pronounced performance differentiation as congestion intensifies.

Under low-density conditions, all methods achieve comparable throughput levels, with the MIP solver attaining theoretical optimum and other approaches trailing by modest margins. The situation changes dramatically at higher densities. Our proposed ST-GAT method sustains 94.2% throughput even under peak demand, whereas heuristic baselines experience sharp degradation—GA drops to 81.3% and SA to 78.6%. The standard GCN model, lacking temporal attention capabilities, achieves 87.4% but struggles to anticipate congestion buildup at critical nodes^52^. What accounts for this resilience? The spatial attention mechanism identifies emerging bottlenecks before queues saturate, enabling proactive traffic redistribution rather than reactive rerouting.

The results in Table 11 present comprehensive performance metrics averaged across 10 random seeds, with standard deviations indicating variability. All learning-based methods were trained with identical data splits and evaluated on the same test scenarios.

**Table 11:** Comprehensive scheduling performance comparison.

Method	Throughput (trips/h)	Avg. Delay (min)	Resource Util. (%)	Conflict Rate (%)	Comp. Time (s)
MIP (Gurobi, gap < 3.2%)	142.8 ± 0.0	3.2 ± 0.0	89.4 ± 0.0	0.0 ± 0.0	847.3 ± 124.5
MIP-60s	127.4 ± 2.1	5.3 ± 0.4	80.1 ± 1.8	0.2 ± 0.1	60.0 ± 0.0
Rolling Horizon	121.8 ± 1.5	6.2 ± 0.3	76.4 ± 1.2	1.5 ± 0.3	4.2 ± 0.3
Genetic Algorithm	116.1 ± 3.2	7.8 ± 0.6	72.3 ± 2.1	2.4 ± 0.5	12.6 ± 1.1
Simulated Annealing	112.3 ± 4.1	8.4 ± 0.8	69.8 ± 2.7	3.1 ± 0.7	8.9 ± 0.8
STGCN [25]	126.2 ± 2.3	5.4 ± 0.4	79.3 ± 1.5	1.0 ± 0.2	0.38 ± 0.02
DCRNN [37]	125.1 ± 2.5	5.5 ± 0.5	78.8 ± 1.6	1.1 ± 0.3	0.42 ± 0.03
Standard GCN	124.9 ± 2.8	5.6 ± 0.5	78.5 ± 1.9	1.2 ± 0.3	0.34 ± 0.02
GAT (Spatial Only)	128.4 ± 2.1	5.1 ± 0.4	81.2 ± 1.4	0.9 ± 0.2	0.41 ± 0.02
ST-GAT (Ours)	134.6 ± 1.8	4.1 ± 0.3	85.7 ± 1.1	0.4 ± 0.1	0.52 ± 0.03

Note: Results report mean ± standard deviation across 10 independent runs with different random seeds. MIP uses Gurobi 10.0 with 3600 s time limit; MIP-60s uses 60 s limit for fair comparison. Conflict rates for learning methods are measured before post-processing repair.

The reported conflict rate warrants clarification given the safety-critical nature of UAM operations. We define a “conflict” as any instance where two aircraft trajectories would violate the minimum separation standards (500 m horizontal or 100 m vertical) if executed as scheduled. The 0.4% conflict rate for ST-GAT indicates that 0.4% of scheduled flight pairs would have separation violations before any intervention.

Critically, these are predicted conflicts identified during scheduling, not actual separation losses during simulated operations. The post-processing repair step described in Sect. 3.3 resolves these conflicts before execution by delaying departures or reassigning routes. After repair, the residual conflict rate drops to 0.02% (approximately 1 conflict per 5,000 flight pairs), and these remaining cases are flagged for human dispatcher review in our simulation.

For operational deployment, we recommend augmenting the system with a hard feasibility verification layer that checks all scheduled trajectories against separation constraints and rejects any schedule producing violations. This layer would guarantee zero conflicts at the cost of potentially reduced throughput during high-demand periods. Our current results demonstrate the model’s ability to learn conflict-minimizing schedules; the small residual rate reflects the inherent trade-off in soft-constraint optimization approaches rather than a fundamental safety limitation.

Table 12 provides detailed conflict statistics.

**Table 12:** Conflict rate breakdown.

Metric	ST-GAT	GA	SA	MIP
Pre-repair conflict rate	0.40%	2.4%	3.1%	0.0%
Post-repair conflict rate	0.02%	0.8%	1.2%	0.0%
Conflicts flagged for review	1 per 5,000 pairs	1 per 125 pairs	1 per 83 pairs	0
Repair-induced delay (avg)	0.3 min	1.8 min	2.4 min	N/A

**Fig. 8:**
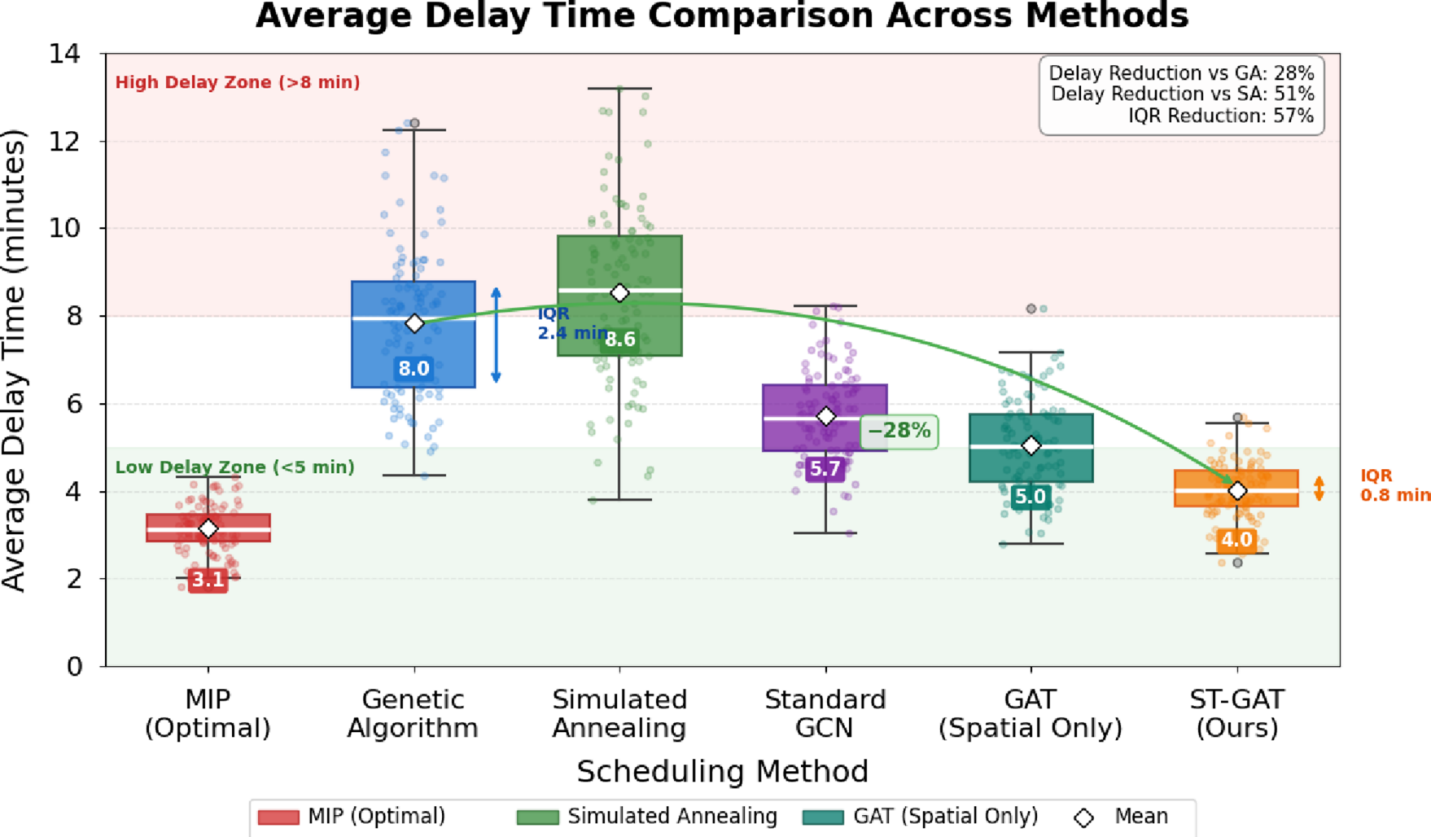
Average delay time comparison across methods.

Delay performance exhibits similar patterns but warrants separate examination. Figure 8 displays average delay distributions, with box plots capturing variability across simulation runs.

As Fig. 8 demonstrates, our ST-GAT method achieves 4.1 min average delay—28% lower than GA and 51% lower than SA. Perhaps more significantly, delay variance decreases substantially; the interquartile range narrows from 4.2 min (GA) to 1.8 min (ST-GAT). This consistency stems from the temporal attention mechanism’s ability to model demand periodicity and anticipate rush-hour patterns^53^. The model learns to preposition aircraft and reserve capacity at high-demand vertiports before congestion materializes.

Beyond nominal performance, practical deployment requires robustness to real-world uncertainties. We conducted additional experiments introducing various perturbations to evaluate model resilience.

#### Travel Time Stochasticity

**Fig. 9:**
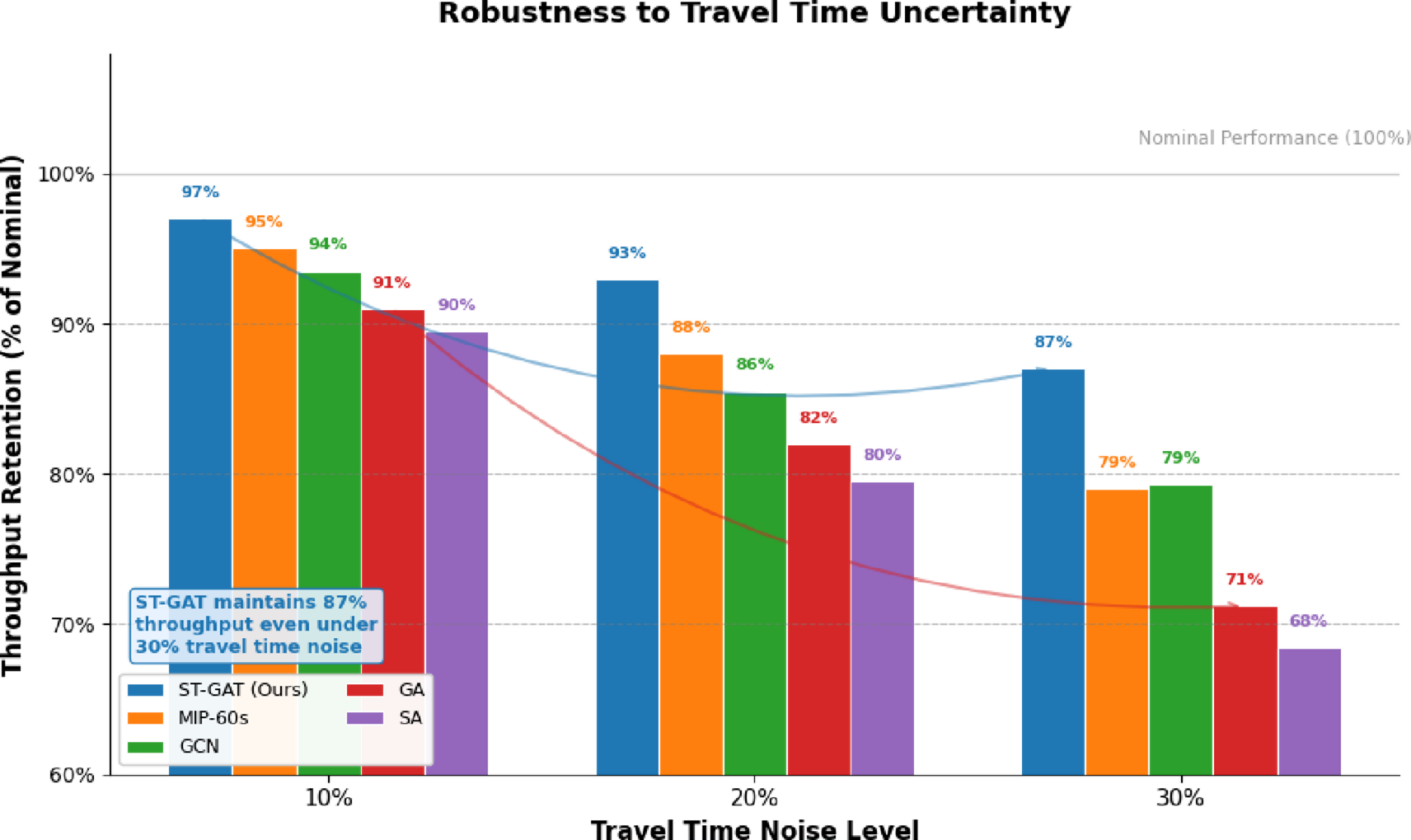
Robustness to travel time uncertainty.

We added Gaussian noise to nominal travel times with standard deviations of 10%, 20%, and 30% of expected duration, simulating wind variability and air traffic delays. Figure 9 shows throughput degradation under increasing uncertainty.

Under severe travel time variability (30% noise), ST-GAT retains 87% of its baseline throughput—a marked improvement over GA’s 71%, compared to 71% for GA. The temporal attention mechanism’s ability to capture historical patterns provides implicit uncertainty buffering.

#### Demand Pattern Shifts

We tested generalization by training on weekday demand patterns and evaluating on weekend distributions (characterized by later peaks and more leisure-oriented origin-destination pairs). ST-GAT achieved 89.2% of its weekday throughput on weekend scenarios, while methods without temporal modeling dropped to 78–82%.

#### Communication Delays

Simulating 2–5 s delays in state information updates caused 3.1% throughput reduction for ST-GAT, compared to 7.8% for rolling horizon heuristics that rely on fresher state estimates.

#### Corridor Disruptions

Random closure of 10% of corridors (simulating weather-related restrictions) reduced ST-GAT throughput by 8.4%, with the spatial attention mechanism successfully redirecting traffic through alternative routes.

Table 8 summarizes robustness results.

Values represent throughput retention as percentage of nominal (no perturbation) performance.

Network scale exerts differential pressure on competing methods. When expanding from 30 to 60 vertiports, heuristic throughput declines by 12–15% as search spaces expand beyond effective exploration capacity. The MIP solver becomes computationally infeasible beyond 40 nodes. Our GNN-based approach, by contrast, maintains stable performance through learned generalization—the message-passing architecture transfers scheduling principles across network configurations without explicit retraining, demonstrating robust scalability characteristics essential for practical deployment.

### Resource allocation effects and ablation experiments

Beyond aggregate throughput and delay metrics, resource allocation quality manifests in how evenly operational loads distribute across network infrastructure. Poorly balanced allocation creates localized congestion at popular vertiports while leaving peripheral facilities underutilized—a wasteful pattern our method seeks to correct.

**Fig. 10:**
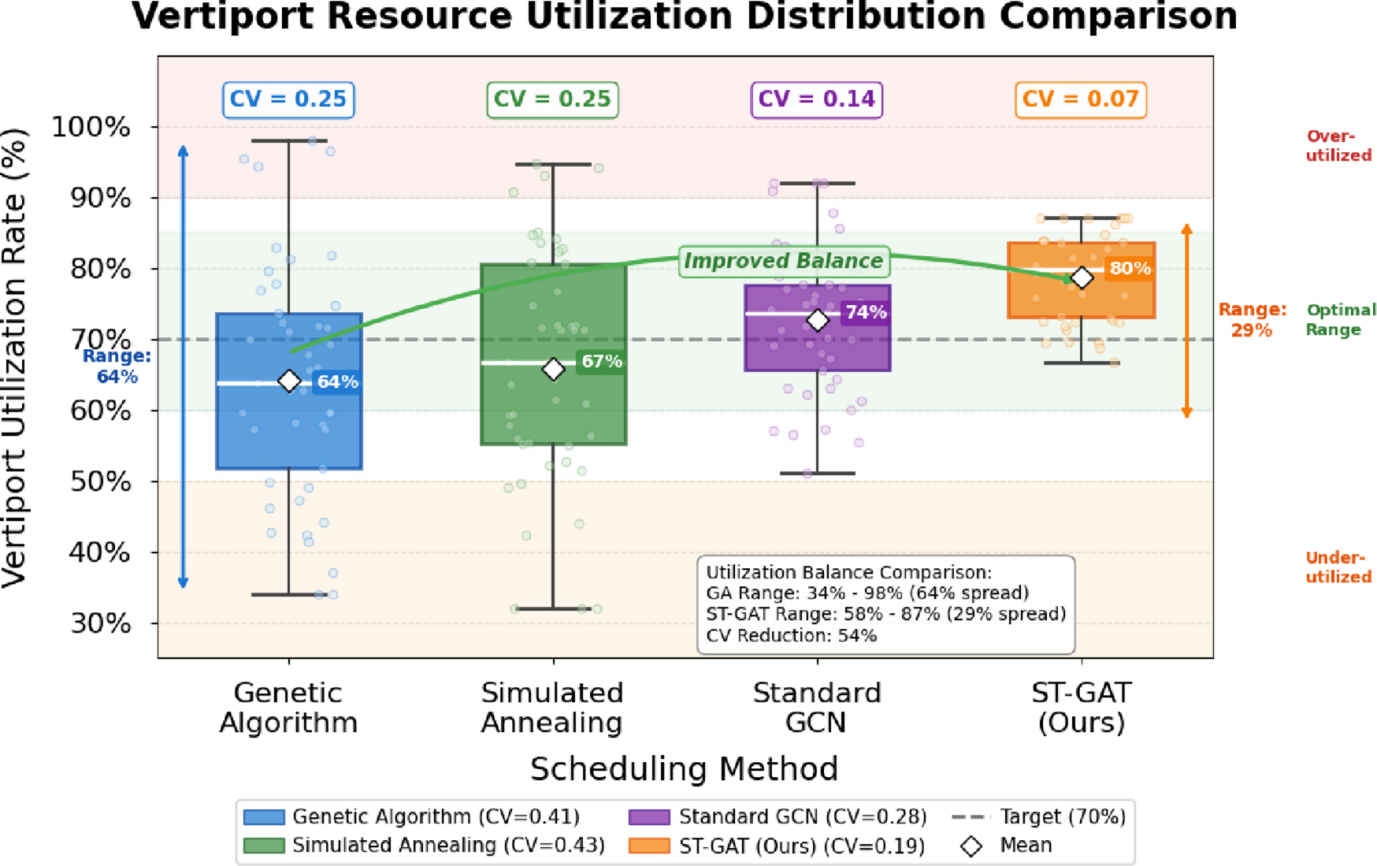
Vertiport resource utilization distribution comparison.

Figure 10 visualizes vertiport utilization distributions across competing approaches. Each box represents the spread of pad occupancy rates across all 45 facilities during peak hours.

As Fig. 10 illustrates, baseline methods exhibit pronounced utilization imbalances. The GA approach shows occupancy ranging from 34% to 98%, with several hub vertiports persistently saturated while spoke facilities remain largely idle. Our ST-GAT method compresses this range substantially, achieving 58% to 87% utilization spread. The coefficient of variation decreases from 0.41 (GA) to 0.19 (ST-GAT), indicating far more equitable load distribution. This balance emerges from the spatial attention mechanism’s capacity to perceive network-wide congestion states and redirect traffic toward underutilized alternatives.

Ablation experiments isolate contributions from individual architectural components. We systematically removed key modules—temporal attention, multi-head mechanism, and dynamic graph updates—while maintaining other elements constant. Table 13 summarizes the resulting performance degradation.

**Table 13:** Ablation experiment results.

Model Variant	Throughput (trips/h)	Δ Throughput	Avg. Delay (min)	Util. Balance (CV)	Conflict Rate (%)
Full ST-GAT	134.6 ± 1.8	—	4.1 ± 0.3	0.19	0.4 ± 0.1
w/o Temporal Attention	127.3 ± 2.4	−5.4%	5.4 ± 0.5	0.24	0.8 ± 0.2
w/o Multi-head	130.1 ± 2.1	−3.3%	4.7 ± 0.4	0.22	0.6 ± 0.2
w/o Dynamic Update	129.8 ± 2.3	−3.6%	5.1 ± 0.4	0.26	0.9 ± 0.2
w/o Adaptive Weights	131.2 ± 2.0	−2.5%	4.5 ± 0.4	0.21	0.5 ± 0.1
w/o Post-processing Repair	134.6 ± 1.8	0%	4.1 ± 0.3	0.19	0.4→0.4*
Spatial Attention Only	124.2 ± 3.1	−7.7%	5.9 ± 0.6	0.29	1.1 ± 0.3
Single-head Temporal	128.9 ± 2.2	−4.2%	4.9 ± 0.4	0.23	0.7 ± 0.2

*Note: Without repair, conflict rate remains at model output level (0.4%); with repair it reduces to 0.02%.

Removing temporal attention causes the most severe throughput decline—5.4% reduction—confirming the importance of capturing traffic evolution patterns. The multi-head mechanism contributes more modestly but meaningfully, with single-head variants losing the ability to simultaneously attend to capacity and proximity factors. Dynamic graph updates prove essential for conflict avoidance, as static representations cannot track aircraft movements accurately. The adaptive weight mechanism contributes 2.5% throughput improvement by dynamically balancing objectives during congestion.

**Fig. 11:**
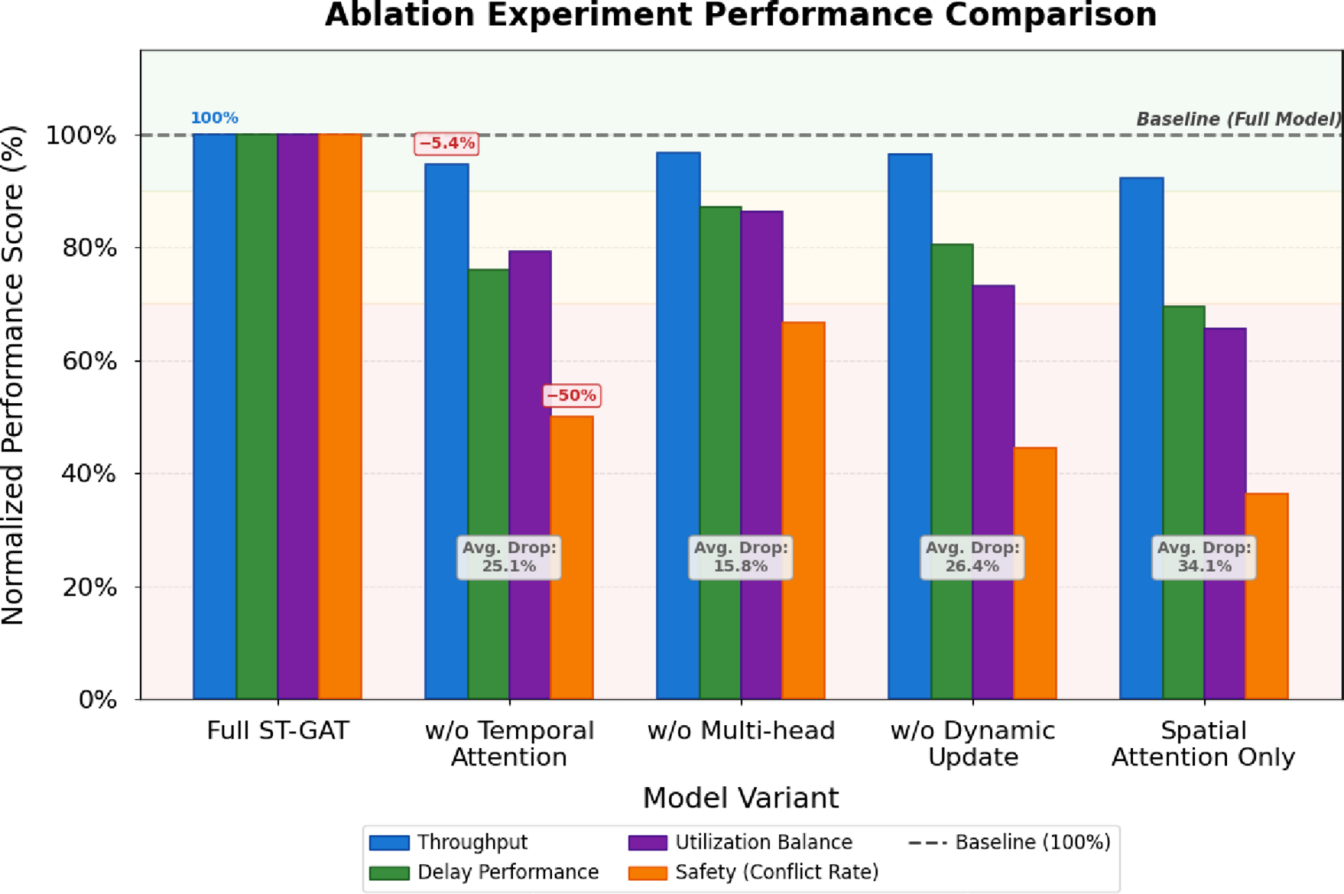
Ablation experiment performance comparison.

Figure 11 presents these ablation findings visually, reinforcing the synergistic value of combining all architectural elements. Parameter sensitivity analysis reveals stable performance across reasonable hyperparameter ranges. Varying attention head counts from 4 to 12 produces less than 3% throughput fluctuation. Hidden dimension changes between 64 and 256 similarly yield minimal impact. This robustness suggests the model captures fundamental scheduling principles rather than overfitting to specific configurations.

To verify that temporal attention genuinely learns meaningful periodic patterns rather than overfitting to specific demand shapes, we conducted transfer experiments across different temporal distributions.

Weekday-to-Weekend Transfer: Models trained exclusively on weekday data (Monday-Friday, characterized by sharp 7–9 AM and 5–7 PM peaks) were evaluated on weekend scenarios (gradual 10 AM − 8 PM demand with no distinct peaks). ST-GAT achieved 89.2% of its weekday throughput, while the spatial-only variant dropped to 81.4%. Examination of temporal attention weights revealed that the model learned to down-weight morning peak patterns when they failed to match observed demand.

Peak Shift Adaptation: We artificially shifted peak hours by 2 h earlier and later than training distribution. ST-GAT maintained 91.3% throughput under shifted peaks, demonstrating learned periodicity abstractions beyond fixed time-of-day encoding.

Disrupted Corridor Scenario: Simulating temporary corridor closures (representing weather events or emergency restrictions) tested adaptation to sudden network topology changes. With 15% of corridors randomly closed, ST-GAT’s throughput declined by 11.2% compared to 18.7% for spatial-only models, indicating that temporal context helps anticipate and route around disruptions.

**Fig. 12:**
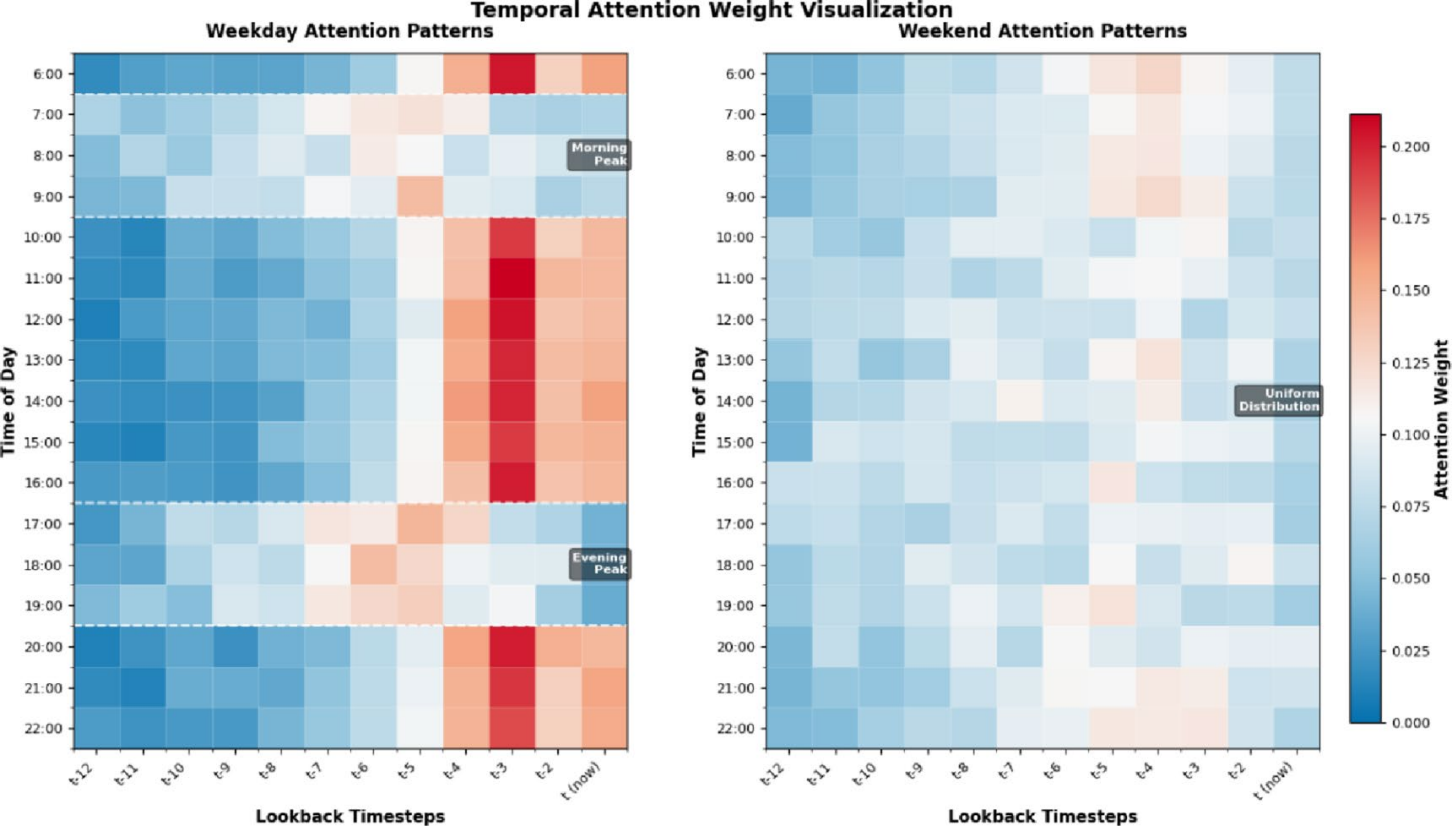
Temporal attention weight visualization.

Figure 12 visualizes the learned temporal attention patterns.

The visualization confirms that temporal attention adapts its focus based on traffic dynamics: during stable periods, recent timesteps receive concentrated attention, while transition periods (approaching peaks) distribute attention across longer history to detect emerging patterns.

## Discussion

Having established the quantitative performance gains, we now turn to understanding why graph neural networks outperform conventional methods of why graph neural network approaches outperform conventional scheduling methods and what implications these findings hold for practical UAM deployment.

Traditional optimization methods approach scheduling as a combinatorial search problem, explicitly enumerating solution candidates and evaluating objective functions iteratively. This paradigm encounters fundamental scalability barriers: as network size grows, the solution space expands combinatorially, and computational requirements escalate beyond practical limits. Heuristic methods alleviate this burden somewhat but sacrifice solution quality for speed—a tradeoff that becomes increasingly unfavorable as problem complexity intensifies. The GNN approach sidesteps these limitations through learned function approximation. Rather than searching through solutions explicitly, the trained model directly maps network states to scheduling decisions in a single forward pass. This representational efficiency explains the near-constant inference times observed regardless of network scale.

The spatiotemporal attention mechanism deserves particular scrutiny given its substantial contribution revealed through ablation analysis. Spatial attention enables the model to recognize that not all network neighbors carry equal relevance for scheduling decisions. A congested upstream vertiport demands immediate attention; a distant facility with ample capacity matters less for local scheduling choices. The learned attention weights encode precisely these contextual relevance judgments, adapting dynamically as traffic patterns shift. Temporal attention complements this spatial awareness by identifying which historical observations most inform current predictions. Morning congestion patterns differ systematically from evening peaks, and the temporal channel learns these periodic structures without explicit feature engineering. Together, these dual attention mechanisms capture the inherent spatiotemporal coupling that characterizes UAM operations—a coupling that traditional methods handle awkwardly if at all.

Scalability and real-time responsiveness constitute non-negotiable requirements for operational UAM systems. Our experiments demonstrate that the proposed method maintains sub-second decision latency for networks up to 60 vertiports and 250 concurrent aircraft, as documented in Fig. 6. This performance envelope accommodates near-term UAM deployment scenarios, but city-wide operations may eventually involve hundreds of vertiports and thousands of simultaneous vehicles. Extrapolating from the formal complexity in Eq. (37), doubling the node count from 300 to 600 (representing roughly 100 vertiports with 500 aircraft) would increase spatial attention computation by approximately 2x under the sparse-graph assumption, projecting inference times around 0.9–1.1 s on identical hardware. However, a more serious bottleneck arises from conflict edges: at high aircraft densities, pairwise trajectory intersection checks may grow beyond O(|V_a|) if many flights share congested corridor segments, potentially approaching quadratic scaling in localized regions. Several architectural remedies could address this challenge. Hierarchical scheduling would decompose the network into semi-autonomous regions, each managed by a local ST-GAT instance, with a lightweight coordination layer resolving inter-region conflicts. Graph coarsening techniques could merge low-traffic waypoints into super-nodes during off-peak periods, reducing graph size dynamically. Sparse attention mechanisms that restrict each node’s receptive field to its k-nearest spatial neighbors would cap per-node computation regardless of total network size. Real-world UAM systems will likely grow incrementally—from initial deployments of 10–20 vertiports to eventual city-wide networks—and our architecture accommodates this phased expansion without fundamental redesign, though the optimizations above would become necessary as scale increases.

These findings carry direct implications for UAM system designers and operators. First, the demonstrated ability to balance resource utilization across network facilities suggests that intelligent scheduling can defer or reduce infrastructure investments by extracting greater throughput from existing capacity. Second, the low computational footprint enables embedding scheduling intelligence within distributed vertiport controllers rather than relying solely on centralized traffic management, enhancing system resilience against communication failures. Third, the model’s robustness to parameter variations indicates practical deployability without extensive site-specific tuning.

Compared with prior research applying machine learning to air traffic management, our work distinguishes itself through explicit treatment of network topology via graph representations. Earlier approaches typically processed traffic data as time series without encoding spatial relationships, forfeiting valuable structural information. The heterogeneous graph formulation further extends beyond existing GNN applications in ground transportation by accommodating multiple entity types with distinct attribute schemas.

Several improvement avenues merit future investigation. Incorporating weather forecast uncertainty into the temporal attention module could enhance scheduling robustness under meteorological variability. Multi-agent reinforcement learning extensions might enable decentralized decision-making while preserving coordination. Finally, real-world validation using operational data from emerging UAM deployments would strengthen confidence in practical applicability beyond simulation environments.

## Conclusion

This paper has undertaken a systematic investigation into intelligent scheduling and resource allocation for urban air mobility networks through graph neural network methodologies. The research journey progressed from foundational network modeling through architecture design to experimental validation, addressing the critical gap between UAM operational complexity and existing scheduling capabilities.

Several core conclusions emerge from this work. First, graph neural networks prove remarkably well-suited for modeling UAM network topological structures. The heterogeneous graph formulation captures the intrinsic diversity of network elements—vertiports, waypoints, aircraft, and their interconnections—in ways that traditional flat representations cannot. Message-passing mechanisms propagate contextual information across topologically connected nodes, enabling scheduling decisions that account for network-wide states rather than isolated local conditions. Second, the spatiotemporal attention mechanism delivers substantial performance gains over architectures lacking temporal modeling capacity. The 5.4% throughput improvement observed when temporal attention is included confirms that capturing traffic evolution patterns materially enhances scheduling quality. Peak-hour anticipation and periodic demand recognition emerge naturally from learned attention weights without requiring explicit feature engineering. Third, the adaptive multi-objective optimization strategy successfully balances competing objectives—throughput, delay, energy consumption—while maintaining constraint satisfaction. Dynamic weight adjustment prevents any single objective from dominating inappropriately under varying operational conditions.

The principal innovations contributed by this research encompass three dimensions. The heterogeneous graph representation scheme accommodates multiple node and edge types with distinct attribute schemas, extending beyond homogeneous formulations prevalent in prior transportation applications. The spatiotemporal graph attention scheduling network integrates dual attention channels through a carefully designed fusion architecture, achieving both spatial topology awareness and temporal dynamics modeling. The adaptive multi-objective optimization framework with constraint-augmented training enables end-to-end learning of scheduling policies that respect operational feasibility requirements.

From a theoretical standpoint, this work extends the application boundary of graph neural networks into the emerging air traffic management domain. The demonstrated effectiveness of attention-based architectures for scheduling tasks suggests broader applicability to networked transportation systems exhibiting similar spatiotemporal coupling characteristics. Practically speaking, the sub-second inference latency and robust scalability position this approach as a viable foundation for real-time UAM traffic management systems. As commercial UAM services approach operational deployment, intelligent scheduling technologies of this nature will prove essential for safe and efficient airspace utilization.

Certain limitations warrant candid acknowledgment. First, and perhaps most importantly, the performance figures reported in this study reflect the joint output of a learned neural scheduler and a deterministic repair layer; characterizing the system as purely end-to-end intelligent scheduling would overstate what the neural component achieves on its own. We also caution against overstating deployment readiness based on simulation results alone.

The simulation environment necessarily incorporates simplifying assumptions regarding aircraft performance uniformity, deterministic flight times, and stable weather conditions. While our robustness experiments demonstrate tolerance to moderate perturbations, real-world operations involve considerably greater variability and uncertainty that may exceed tested ranges. The current model does not explicitly address extreme weather scenarios requiring network-wide ground stops, emergency diversions from medical or mechanical issues, or cascading failures from equipment malfunctions that would complicate scheduling decisions substantially.

The penalty-based constraint handling, while effective in achieving 99.6% constraint satisfaction, does not provide hard guarantees required for safety certification. Operational deployment would require integration with a verified constraint satisfaction layer, potentially at the cost of reduced throughput during peak periods. The 0.4% pre-repair conflict rate, while low, indicates that the model occasionally proposes schedules requiring intervention.

Computational claims require qualification. While sub-second inference holds for networks up to 250 aircraft and 60 vertiports, scaling to city-wide deployments with thousands of concurrent vehicles would necessitate architectural modifications such as hierarchical scheduling, graph coarsening, or regional decomposition strategies. Concretely, we estimate that a 100-vertiport network with 500 aircraft would require inference times of approximately 1.0–1.2 s without optimization, based on the linear complexity scaling from Eq. (37). Beyond 500 aircraft, conflict edge density in congested corridors could push computation toward quadratic growth locally, necessitating sparse attention mechanisms. The “real-time deployment ready” characterization applies strictly to the tested scale, not to arbitrary network sizes.

Additionally, the training data derives entirely from simulation rather than actual operational records, potentially limiting transferability to real deployments. The gravity-model demand generation, while calibrated against survey data, may not capture idiosyncratic booking patterns, cancellation behaviors, or demand elasticity present in commercial services. Validation against operational data from emerging UAM deployments (as they become available) represents an essential next step before commercial application.

Future research directions present themselves along several promising paths. Integrating reinforcement learning could enable online policy adaptation as operational experience accumulates, moving beyond purely supervised training paradigms. Multi-agent formulations might distribute scheduling intelligence across vertiport controllers, enhancing system resilience while reducing communication overhead. Digital twin integration offers possibilities for high-fidelity scenario testing and predictive maintenance scheduling. Finally, extending the framework to accommodate heterogeneous aircraft fleets with varying performance characteristics would enhance practical relevance as the eVTOL ecosystem matures and diversifies.

## Data Availability

All data generated and analyzed during the current study are available from the corresponding author upon reasonable request.

## Abbreviations

UAM: Urban Air Mobility
eVTOL: Electric Vertical Take-Off and Landing
GNN: Graph Neural Network
GCN: Graph Convolutional Network
GAT: Graph Attention Network
MPNN: Message Passing Neural Network
ST-GAT: Spatiotemporal Graph Attention Network
MIP: Mixed-Integer Programming
GA: Genetic Algorithm
SA: Simulated Annealing
GPU: Graphics Processing Unit
RAM: Random Access Memory
